# New Cu(II), Cu(I) and Ag(I) Complexes of Phenoxy-Ketimine Schiff Base Ligands: Synthesis, Structures and Antibacterial Activity

**DOI:** 10.3390/molecules30091893

**Published:** 2025-04-24

**Authors:** Miriam Caviglia, Zhenzhen Li, Carlo Santini, Jo’ Del Gobbo, Cristina Cimarelli, Miao Du, Alessandro Dolmella, Maura Pellei

**Affiliations:** 1 School of Science and Technology, Chemistry Division, University of Camerino, Via Madonna Delle Carceri (ChIP), 62032 Camerino, Italy; miriam.caviglia@unicam.it (M.C.); zhenzhen.li@unicam.it (Z.L.); carlo.santini@unicam.it (C.S.); jo.delgobbo@unicam.it (J.D.G.); cristina.cimarelli@unicam.it (C.C.); 2 College of Material and Chemical Engineering, Institute of New Energy Science and Technology, Zhengzhou University of Light Industry, Zhengzhou 450001, China; 3 Department of Pharmaceutical and Pharmacological Sciences, University of Padova, Via Marzolo 5, 35131 Padova, Italy

**Keywords:** silver, copper, Schiff bases, phosphanes, single-crystal XRD, antibacterial activity

## Abstract

Two phenoxy-ketimines ligands, 2-(1-(benzylimino)ethyl)phenol (HL^BSMe^) and 2-((benzylimino)(phenyl)methyl)phenol (HL^BSPh^), were synthesized and used as supporting ligands of new copper(II), copper(I), and silver(I) complexes. In order to confer different solubility properties to the metal complexes and to stabilize Cu and Ag in their +1 oxidation state, the lipophilic triphenylphosphine (PPh_3_) and the hydrophilic 1,3,5-triaza-7-phosphaadamantane (PTA) were selected as co-ligands in the syntheses of the Cu(I) and Ag(I) complexes. All compounds were characterized by CHN analysis, NMR, FT-IR spectroscopy, and electrospray ionization mass spectrometry (ESI-MS); the molecular structure of the copper(II) complex [Cu(L^BSPh^)_2_] was also determined by single-crystal X-ray diffraction. Finally, the antibacterial activity of the metal complexes, the Schiff base ligands and phosphane co-ligands, were assessed by determining the minimum inhibitory concentration (MIC) and minimum bactericidal concentration (MBC) against Gram-negative (*Escherichia coli*) and Gram-positive bacteria (*Staphylococcus aureus*).

## 1. Introduction

In recent years, the phenomenon of antibiotic resistance in hospitals, communities, and the environment has increasingly grown. Antibacterial resistance is a major global public health challenge, associated with an estimated 4.95 million deaths in 2019 [1,2]. Due to these developments, the World Health Organization (WHO) published the first list of drug-resistant bacterial priority pathogens classified as critical in 2017 [3]. Building on the 2017 edition, in 2024, the WHO published an updated list of drug-resistant bacteria posing the greatest threat to human health, aiming to guide the development of new treatments and strategies to prevent and mitigate the spread of antimicrobial resistance (AMR) [2]. Despite the current work, the global antibiotic pipeline is marked by limited innovation and limited global access to both new and existing treatments. This increase in antimicrobial resistance and the misuse of antimicrobials have led to the need to develop new antimicrobial compounds [4,5,6,7,8].

Medicinal inorganic chemistry offers possibilities for the design of therapeutic agents not readily available to organic compounds [7,9,10]. A successful strategy in antimicrobial chemotherapy has been the use of metallo-drugs, and this strategy has the potential to be used for treating multidrug-resistant infections more efficiently [4,11,12]. As a class of molecules, Schiff bases have been a topic of considerable interest, owing to their versatile metal chelating properties, and have been used to coordinate almost all metal ions [13,14,15,16,17,18,19,20,21,22,23,24]. Schiff bases and their metal complexes have been reported to exhibit a wide range of biological activities [15,25,26,27,28,29,30,31,32,33,34], including anticancer [35,36] and antimicrobial properties [37,38,39,40,41,42,43,44,45]. In most cases, the metallic derivatives of Schiff bases were found to exhibit higher antimicrobial activities than their parent ligands [27,46].

Numerous Schiff bases and their copper(II) derivatives have been reported to possess promising catalytic [47,48], anticancer [49,50,51,52], or biological properties [41,45,53,54,55,56,57,58,59,60,61] and have been successfully used as models in biological systems [62]. In particular, several Cu(II) complexes of *N,O*-donor Schiff base ligands were found to exhibit favorable antimicrobial activities [57,63,64,65,66,67,68,69,70,71].

Despite the enormous amount of work devoted to the synthesis and characterization of copper(II) Schiff bases complexes [65], there are relatively few reports devoted to the corresponding Cu(I) complexes, perhaps due to their tendency to undergo disproportionation to copper metal and copper(II) compounds in the absence of stabilizing co-ligands [72,73,74]. Schiff bases as chelating N-donors and phosphanes as P-donors were recently employed for the preparation of photoactive neutral [75,76] or cationic heteroleptic Cu(I) complexes [77,78,79]. On the other hand, in view of the wide spectra of antimicrobial activities against bacteria and fungi showed by silver(I) [80] and related to metal complexes [81,82,83], plenty of studies focused on the antimicrobial properties of silver(I) complexes with Schiff bases [84,85]. Recently, the Schiff bases Cu(I) and Ag(I) complexes incorporating phosphanes as co-ligands have awakened a new interest towards their biological properties [86].

**Scheme 1:**
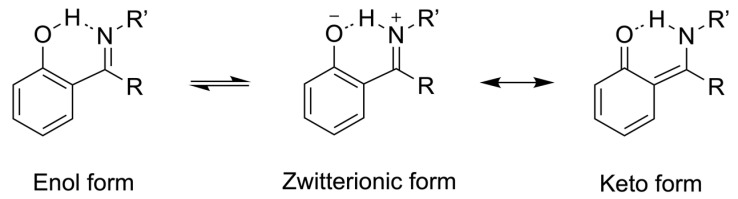
Tautomerism of 2-imidoylphenols.

Among the various types of Schiff bases [87,88,89,90,91,92,93,94,95,96], the phenoxy-imines and phenoxy-ketimines are of particular interest. These 2-imidoylphenols can exist in tautomeric forms due to intramolecular H-bonding between the hydroxyl H-atom and the N-atom (Scheme 1) [97,98,99].

Depending on the extent of the interaction, complete transfer of the proton from the hydroxyl group to the nitrogen atom may occur, giving conversion of the molecular form (benzenoid, enol-type) into the quinoid, keto-type tautomer [98,100]. Zwitterionic species characterized by an intramolecular charge-separation are possible intermediates in the tautomerization process [99]. The replacement of the hydrogen atom by the methyl, ethyl, or phenyl substituents in the C-C(H)=N moiety in phenoxy-imines Schiff bases shortens the intramolecular hydrogen bonds in related phenoxy-ketimines [101].

In recent years, metal complexes of *N*,*O*-bidentate phenoxy-ketimines Schiff base ligands have attracted much attention from researchers [47,48,102,103,104,105,106,107,108,109,110]. However, to our knowledge, copper(I)- and silver(I)-based complexes supported by *N*,*O*-bidentate phenoxy-ketimines, including phenoxy-imines Schiff base ligands, remain an unexplored research field.

**Scheme 2:**
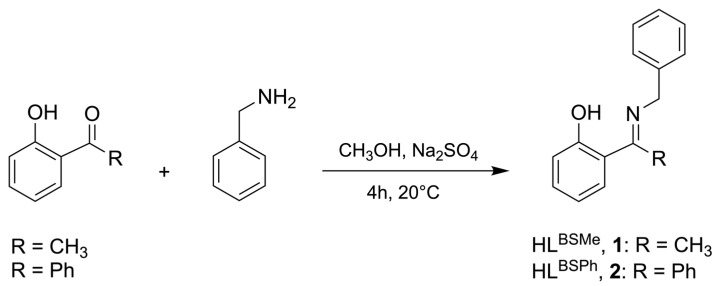
Synthesis of HL^BSMe^ (**1**) and HL^BSPh^ (**2**) ligands.

**Scheme 3:**
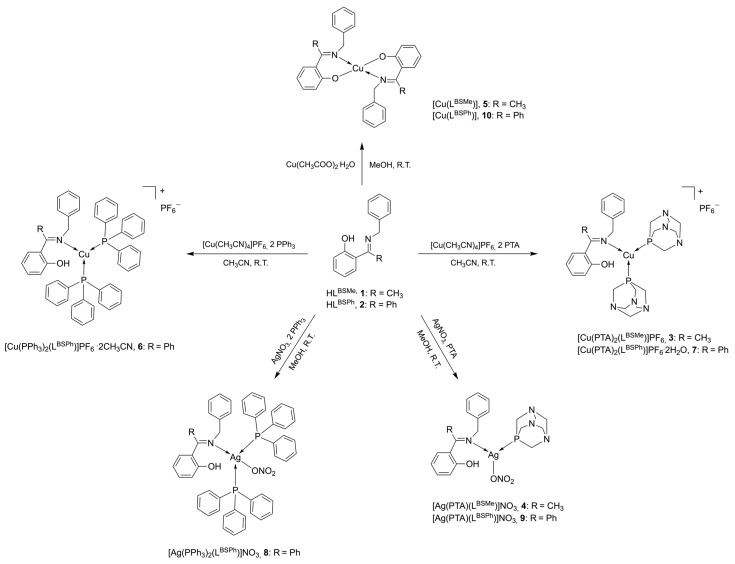
Synthesis of compounds **3**–**10**.

Therefore, as part of our continuous investigation on the chemical and biological properties of copper- and silver-containing coordination compounds [111,112,113,114,115,116,117], we report here the syntheses, characterization, and biological evaluation of new Cu(II), Cu(I), and Ag(I) complexes containing phosphanes and the phenoxy-ketimines ligands, 2-(1-(benzylimino)ethyl)phenol (HL^BSMe^) (Oletimol), and 2-((benzylimino)(phenyl)methyl)phenol (HL^BSPh^) (Scheme 2 and Scheme 3). The ligands were selected to modify the steric and electronic properties of the resulting metal complexes using phenyl and methyl groups on the C-C(R)=N imino moiety, respectively. In designing the novel phenoxy-ketimines metal complexes, the lipophilic triphenylphosphine (PPh_3_) and the hydrophilic 1,3,5-triaza-7-phosphaadamantane (PTA) were selected as co-ligands, in order to stabilize copper and silver in their +1 oxidation state and to confer different solubility properties to the corresponding metal complexes.

The antimicrobial properties of the new copper and silver complexes as well as of the corresponding uncoordinated ligands and co-ligands were evaluated against Gram-negative (*Escherichia coli*) and Gram-positive (*Staphylococcus aureus*) bacteria. The antimicrobial data have been compared with the control antibiotic ciprofloxacin to assess their relative efficacy [66].

## 2. Results and Discussion

### 2.1. Synthesis and Characterization

The phenoxy-ketimines ligands HL^BSMe^ and HL^BSPh^ were prepared using a modified literature method [118,119,120], by the condensation reaction of the 2-acylphenols 2′−1-(2-hydroxyphenyl)ethan-1-one or (2-hydroxyphenyl)(phenyl)methanone, respectively, with stoichiometric amounts of benzylamine in methanol, and isolated as yellow solids in very good yield and high purity (Scheme 2).

The ligands were fully characterized by ^1^H-NMR (Table S1), ^13^C-NMR, FT-IR spectroscopy, and ESI-MS spectrometry. Both 1,3,5-triaza-phosphaadamantane copper(I) complexes [Cu(HL^BSMe^)(PTA)_2_]PF_6_ (**3**) and [Cu(HL^BSPh^)(PTA)_2_]PF_6_·2H_2_O (**7**) were synthesized in CH_3_CN using, as starting materials, the ligand HL^BSMe^ or HL^BSPh^, the metal acceptor [Cu(CH_3_CN)_4_]PF_6,_ and the PTA co-ligands in the stoichiometric ratio 1:1:2, respectively (Scheme 3). Analogously, triphenylphosphine copper(I) complex [Cu(HL^BSPh^)(PPh_3_)_2_]PF_6_·2CH_3_CN (**6**) was synthesized in CH_3_CN using, as starting material, the ligand HL^BSPh^, the metal acceptor [Cu(CH_3_CN)_4_]PF_6,_ and the PPh_3_ co-ligands in the stoichiometric ratio 1:1:2 (Scheme 3).

Elemental analyses and spectroscopic studies such as FT-IR, ^1^H-, ^31^P-NMR, and ESI-MS confirm the stoichiometry of the synthesized complexes **3**, **6**, and **7**. All the expected absorption bands were observed in the FT-IR spectra. The most characteristic feature of the complexation is the removal of the O⋯H⋯N hydrogen bond [101,121] and the presence of a very broad ν(OH) absorption band at about 3200 cm^−1^. The complexes exhibit weak bands in the range of 2878–3070 cm^−1^ typical of C-H stretching. The strong absorptions at 1571–1618 cm^−1^ are due to the stretching of the aromatic and imine bonds (C=C and C=N), while very intense absorptions in the range of 831–832 cm^−1^ are attributable to the stretching of the PF_6_^−^ counterion. The ^1^H-NMR spectra, recorded in CD_3_CN for complexes **3**, **6**, and **7**, confirm the stoichiometric ratio between the Schiff base ligand and phosphane co-ligands. They showed a single set of resonances for the skeletal ligands, indicating that the Schiff base ligand protons are equivalent. The signals of phenolic O*H* protons appeared as broad peaks in the range of 15.41–16.24 ppm, the signal of the C*H*_2_Ph protons is visible at δ 4.55–4.83 ppm, while the signal of the ketimine methyl C*H*_3_C=N proton for complex **3** appeared at δ 2.47 ppm, with a slight shift with respect to the signal of the free ligand due to the coordination to the copper acceptor. The aromatic hydrogens of ligand and triphenylphosphine co-ligands for [Cu(HL^BSPh^)(PPh_3_)_2_]PF_6_·2CH_3_CN (**6**) are detectable in the range of 6.71–7.69 ppm, while in the spectra of [Cu(HL^BSMe^)(PTA)_2_]PF_6_ (**3**) and [Cu(HL^BSPh^)(PTA)_2_]PF_6_·2H_2_O (**7**) recorded in CD_3_CN, the NC*H*_2_P protons of PTA co-ligands are visible as singlets at δ 4.06–4.09 ppm and the related NC*H*_2_N protons show characteristic AB quartets in the range of 4.52–4.60 ppm.

The ^31^P-NMR spectrum of [Cu(HL^BSPh^)(PPh_3_)_2_]PF_6_·2CH_3_CN (**6**) performed in CD_3_CN gives a broad singlet peak at −0.30 ppm, downfield shifted with respect to the value of the free triphenylphosphine in the same solvent (δ = −4.85 ppm). Likewise, a broad singlet is visible in the spectra of the PTA complexes **3** and **7**, recorded in CD_3_CN, at −91.64 and −89.66 ppm, downfield shifted with respect to the value of the free PTA in CD_3_CN (δ −102.07 ppm). Moreover, the spectra of complexes **3**, **6**, and **7** show the distinctive septets due to the presence of the PF_6_^−^ counterion at about −144.60 ppm with J_F−P_ = 706 Hz. ^1^H-NMR and ^31^P{^1^H}-NMR data of complexes **3**, **6**, and **7** are summarized in Table S1. The ESI-MS study was performed by dissolving complexes **3**, **6**, and **7** in acetonitrile and recording the spectra in ion-positive and ion-negative modes. In the ESI-MS(+) spectrum of **3**, the major peak at *m*/*z* 445 can be attributed to the species [Cu(HL^BSMe^)(PTA)]^+^. Similarly, the ESI-MS(+) spectrum of **7** showed at *m*/*z* 507 the peak due to the species [Cu(HL^BSPh^)(PTA)]^+^, confirming the formation and the stability of the complexes.

The copper(II) complexes [Cu(L^BSMe^)_2_] (**5**) and [Cu(L^BSPh^)_2_] (**10**) were synthesized using the copper(II) acetate salt Cu(CH_3_COO)_2_ and the ligands HL^BSMe^ and HL^BSPh^, respectively, in the stoichiometric ratio 1:2 (Scheme 3). The reaction was performed in methanol at reflux for the synthesis of complex **5** and in acetonitrile at room temperature for the synthesis of **10**. Isolated complexes are air-stable, soluble in CH_3_OH, CH_3_CN, CH_2_Cl_2_, CHCl_3_, and DMSO, and they have been characterized by means of FT-IR spectroscopy and ESI-MS spectrometry. Successful complexation was indicated by a slight shift of the imine bonds (ν(C=N)) in the IR spectra. In particular, the imine and aromatic bands in complexes **5** and **10** are observed at 1588–1597 cm^−1^ and 1569–1599 cm^−1^, respectively, whereas in the corresponding ligand precursors HL^BSMe^ and HL^BSPh^, the bands appear at 1572–1610 and 1563–1604 cm^−1^, respectively. The ESI-MS spectra of complexes **5** and **10** were performed in CH_3_CN solution. In the ESI-MS(+) of complex **5**, peaks at *m*/*z* 287 and 329 are due to the species [Cu(L^BSMe^)]^+^ and [Cu(L^BSMe^) + CH_3_CN]^+^, respectively; the major peaks at *m*/*z* 511 and 576, respectively, are due to the adducts [Cu(L^BSMe^)_2_ + H]^+^ and [Cu(L^BSMe^)_2_ + Na + CH_3_CN]^+^, respectively, confirming the presence of two ligands that coordinate copper. For complex **10**, signals at *m*/*z* 349 and 390 are attributable to the fragments [Cu(L^BSPh^)]^+^ and [Cu(L^BSPh^) + CH_3_CN]^+^.

The silver complexes [Ag(HL^BSMe^)(PTA)]NO_3_ (**4**) and [Ag(HL^BSPh^)(PTA)]NO_3_ (**9**) were synthesized by a treatment of AgNO_3_ with one equivalent of 1,3,5-triaza-phosphadaamantane (PTA) and one equivalent of the corresponding HL^BSMe^ or HL^BSPh^ ligands in methanol and acetonitrile solution, respectively. Likewise, the triphenylphosphine silver(I) complex [Ag(HL^BSPh^)(PPh_3_)_2_]NO_3_ (**8**) was synthesized in CH_3_CN using as starting material the ligand HL^BSPh^, the metal acceptor silver nitrate, and the PPh_3_ co-ligands in the stoichiometric ratio 1:1:2. Isolated complexes are air-stable and have been characterized by means of ^1^H-, ^31^P-NMR and FT-IR spectroscopy, as well as ESI-MS spectrometry. In IR spectra, a slight shift of the imine bonds (ν(C=N)) was observed, indicating complexation. Complexes **4**, **8**, and **9** exhibit a series of bands in the 1479–1292 cm^−1^ region of the IR spectrum that can be attributed to the stretching modes of the NO_3_ group [122,123]. These bands are consistent with those previously reported for analogous silver(I) phosphane complexes [124,125,126]. However, the multiplicity of the observed bands prevents an unambiguous assignment of the nitrate group’s coordination mode—whether monodentate or bidentate—to the metal center.

The ^1^H-NMR spectra of **4**, **8**, and **9** in DMSO-d_6_ showed a set of resonances in agreement with the proposed structure. The slightly shifted resonance at δ 15.45–16.38 ppm corresponds to the OH phenolic proton. In the spectra **4** and **9**, the NC*H*_2_P protons of PTA co-ligands are visible as singlets at δ 4.15–4.16 ppm and the related NC*H*_2_N protons show characteristic AB quartets in the range of 4.40–4.58 ppm. In the spectrum of complex **8**, the aromatic hydrogens of the ligand and triphenylphosphine co-ligands are detectable in the range 6.71–7.63 ppm. In the ^31^P-NMR spectra of the PTA complexes **4** and **9** recorded in CDCl_3_, a singlet is visible at −93.60 and −94.43 ppm, downfield shifted with respect to the value of the free PTA (δ −102.50 ppm). Broad signals are also in the spectra recorded in CD_3_OD and CD_3_CN at 233K, indicating the impossibility to stop or slow the phosphane exchange process, to show doublets in which the coupling of ^31^P to the ^107^Ag and ^109^Ag are resolved. In the ^31^P-NMR spectrum of the triphenylphosphine complex **8** recorded in CDCl_3_ at room temperature, a very broad singlet is visible at 14.68 ppm, downfield shifted with respect to the value of the free PPh_3_ (δ −5.34 ppm). In the spectrum of **8** recorded in CD_3_CN at 233K, a doublet was observed. The related *J*(Ag-^31^P) coupling constant (478 Hz) is of the same order of magnitude of those reported for analogous silver(I) bis(triphenylphosphine) species [111,127,128,129,130]. ^1^H-NMR and ^31^P{^1^H}-NMR data of complexes **4**, **8**, and **9** are summarized in Table S1. The ESI-MS study was performed by dissolving complexes **4**, **8**, and **9** in methanol or acetonitrile and recording the spectra in ion-positive and ion-negative modes. In the ESI-MS(+) spectrum of **4** and **9**, the peaks at *m*/*z* 491 and 553 can be attributed to the species [Ag(HL^BSMe^)(PTA)]^+^ and [Ag(HL^BSPh^)(PTA)]^+^, respectively, confirming the formation and the stability of the complexes.

### 2.2. X-Ray Crystallography

**Figure 1:**
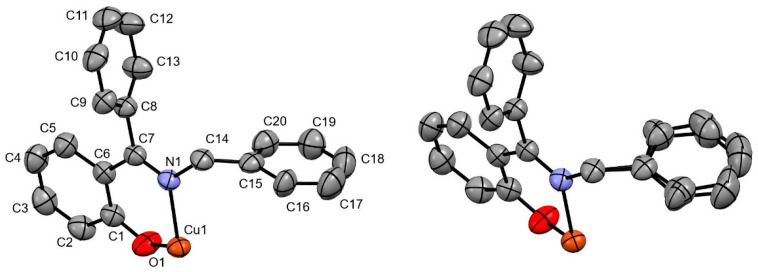
(**Left**): a drawing of the asymmetric unit of [Cu(L^BSPh^)_2_] (**10**), showing the numbering scheme; disorder not shown. (**Right**): same view highlighting the disorder of the phenyl ring C15/C20. Hydrogen atoms omitted; thermal ellipsoids at 50% probability.

Despite several efforts, we were able to get crystalline samples of only one of the aforementioned compounds. In particular, the structure of the [Cu(L^BSPh^)_2_] complex (**10**) was determined by single-crystal diffraction analysis on an item extracted from a batch of very good, dark green prismatic specimens, obtained by a slow recrystallization of the compound from a toluene solution. The investigation revealed that the neutral complex crystallizes in the monoclinic system; the solid-state structure was solved in the centrosymmetric *I*2/*a* space group (No. 15). The asymmetric unit (Figure 1) consists of one half of the molecule, that is, the copper ion, and a single molecule of the anionic 2-(benzylimino(phenyl)methyl)phenolate (L^BSPh^) ligand, which chelates the central Cu atom through the O, N atoms. The phenyl ring of the benzylimino residue of the ligand (C15/C20) is disordered over two possible arrangements; the alternate positions of disordered atoms have been named with a final ‘A’.

Upon chelation, the ligand and the metal form a six-membered metallacycle (Cu1, O1, C1, C6, C7, N1), showing an *envelope* conformation, with the Cu1 atom ‘at the flap’ and departing by 0.55 Å from the mean plane (p1) of the other five atoms, that are instead coplanar within 0.05 Å, given the *sp*^2^ nature of the C6-C7-N1 atoms of the ligand. In the chelating L^BSPh^ molecule, the mean plane of the phenol ring (C1/C6) is almost coplanar with p1 (angle of 1.39°); instead, the mean planes passing through the phenyl rings of the benzyl moiety (C15/C20 or C15A/C20A, respectively, p2 and p2′) or emerging from the methine bridge (C8/C13, p3) are approximately orthogonal to the p1 plane, making with the latter angles of 79.1° (79.6° for p2′), and 75.2°, respectively. The p3 and p2 planes are also roughly orthogonal with respect to each other (angles of 69.9° and 72.2° for p2 and p2′, respectively).

**Figure 2:**
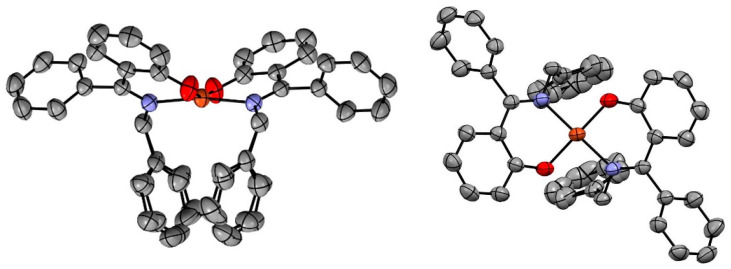
Two orthogonal views of the [Cu(L^BSPh^)_2_] complex. (**Left**): overall view of the complex looking through the coordination plane, highlighting its calix shape; (**right**): view of the Cu(II) coordination plane. Hydrogen atoms omitted; thermal ellipsoids at 50% probability.

The copper coordination environment is completed by a second L^BSPh^ molecule (Figure 2), in which both the O1 and N1 atoms are *trans*-positioned to their symmetry-generated counterparts. The [Cu(L^BSPh^)_2_] complex (**10**) has a calix shape when observed through the O_2_N_2_ coordination plane and the four coordinating atoms around the central copper atom define a moderately distorted square planar environment (τ_4_ and τ_4_′ indexes of 0.21 and 0.18, respectively) [131,132], with O1-Cu1-O1* and N1-Cu1-N1* (* = 3/2 − *x*, *y*, 1 − *z*) angles of 160.77(9)° and 170.07(7)°, respectively—the first quite far from the ideal 180° value. Apparently, the departure is due to the engagement of the O1 atom in an intermolecular non-canonical hydrogen bond with the H18 atom of a nearby phenyl ring (see further below).

With respect to bond distances and angles, the 2-(benzylimino(phenyl)methyl)phenolate ligand does not show unusual metric values when compared to existing data. An investigation in the CCDC database [133] for molecules showing the benzyl(diphenylmethylene)amine moiety returned about 50 entries. A comparison of bond distances next to the C=N link with those found in this work showed that the object with the best overall similarity, that is, with the smaller sum of deviations for examined distances, is the (already reported) ligand itself [121], besides few other compounds [134,135]. Interestingly, molecules in which the C=N distance is close to the one reported here (1.299(2) Å) are those in which a phenolic moiety was also present [121,136,137,138]. The C=N bond participates in some degree of conjugation with the nearby phenyl rings, in known compounds as well as in the present complex. In fact, the C6-C7 and C7-C8 bonds are a little shorter than the ideal 1.54 Å (1.4654(19) and 1.5009(19) Å, compared with mean values of 1.494 and 1.492 Å for known molecules); the same is true for the N1-C14, C14-C15 distances in the benzyl residue (1.4787(17), 1.483(10) Å against corresponding mean values of 1.450 and 1.517 Å in reported compounds). These data suggest that bond conjugation in the ligand is little affected by coordination.

A second search in the CCDC repository was made to seek transition metal complexes showing a ligand similar to L^BSPh^. The search returned a very limited number of Mn, Co, Ni, and Cu compounds showing a tetradentate dimeric form of the L^BSPh^ ligand, all prepared by the same research group [139,140,141,142,143]; to the best of our knowledge, then, **10** is the first Cu(II) complex of the L^BSPh^ ligand reported to date. In known molecules, the O and N donor atoms are always *trans* to each other, opposite to what happens in the [Cu(L^BSPh^)_2_] complex. The Metal-O, Metal-N distances in these molecules fall in the ranges 1.82–1.91 Å, 1.85–2.03 Å, respectively, with differences basically due to the ionic radii of the elements. In fact, the mean values for the Cu-O, Cu-N distances in the Cu(II) known complexes [140] (1.879 and 1.949 Å, respectively) are in very good/good agreement with the 1.8839(11) and 1.9773(12) Å found here. A complete listing of bond lengths and angles for complex **10** is provided in the supporting information (Tables S2 and S3).

**Figure 3:**
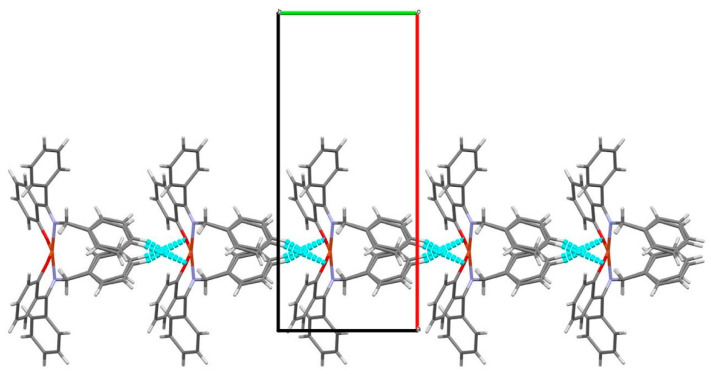
Nonbonding contacts for [Cu(L^BSPh^)_2_]; projection down the crystallographic *c* axis, highlighting the one-dimensional motif generated by the O1····H18, O1····H18A hydrogen bonds (in cyan) and propagating along the crystallographic *b* axis. The π····π pairing of the C15/C20, C15A/C20A rings with the corresponding symmetry-generated counterparts is also visible. Other contacts omitted for clarity.

The examination of relevant nonbonding interactions (metric details listed in Table 1), mostly hydrophobic contacts, shows that the complex units are well packed, and that the unit cell does not show solvent accessible voids. Looking at the asymmetric unit (one half of the complex), just one intermolecular hydrogen bond connects H18 (or H18A) with the O1 atom of a nearby molecule at 3/2 − *x*, −1 + *y*, 1 − *z* (at 2.81/2.85 Å, respectively); this interaction also shows in the symmetry-generated half of the molecule at 1/2 − *x*, *y*, 1 − *z* (Figure 3; further packing diagrams highlighting the other contacts described here can be found in Figures S34–S36 of supporting information). Incidentally, the two symmetry-related halves of the complex have their C15/C20 (or C15A/C20A) rings in roughly eclipsed positions, so they are also coupled by somehow weak π····π interactions, with distances between planes encompassing the alternate arrangements of the rings of 4.20, 4.03 Å, respectively, (compared with 3.35 Å in graphite).

The H18····O1 contact creates a one-dimensional motif which propagates along the crystallographic *b* axis, and it is coupled to a second orthogonal chain due to the C-H····π contacts made by C1/C2 atoms and the H12 atom of another unit at 1 − *x,* 1 − *y,* 1 − *z* (Figure S36). The two chains cross-link each other and create a 2D grid in the *ab* plane (Figure S37). Finally, a third chain is formed by a second, T-shaped C-H····π contact involving the C15/C20 (or C15A/C20A) ring with the H11 atom of a nearby molecule at 1 − *x*, −1/2 + *y*, 1/2 − *z* (Figure S38; only C15····H11 approach shown). The H11 atom in fact points almost straight towards the C15/C20 ring centroid, with a contact length of 2.78 Å (also towards the C15A/C20A ring) and C11-H11-ring centroid angle of 168.8° and 165.0° for C15/C20, C15A/C20A rings, respectively. This chain propagates along the [011] plane and diagonally intersects the above described 2D motif, thus sealing a full 3D contact network.

### 2.3. In-Vitro Antibacterial Activity

**Figure 4:**
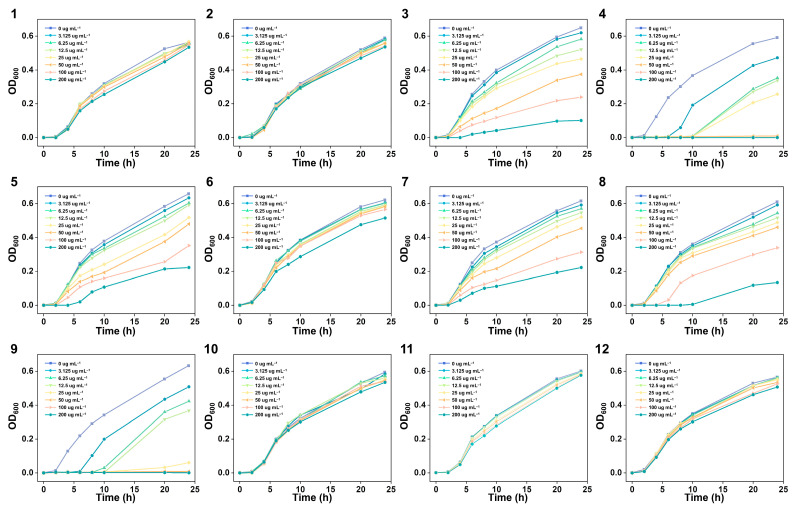
Bacterial growth curves of *E. coli* after treatment with Schiff base ligands (**1** and **2**), metal complexes (**3**–**10**), and phosphane co-ligands (**11** and **12**).

**Figure 5:**
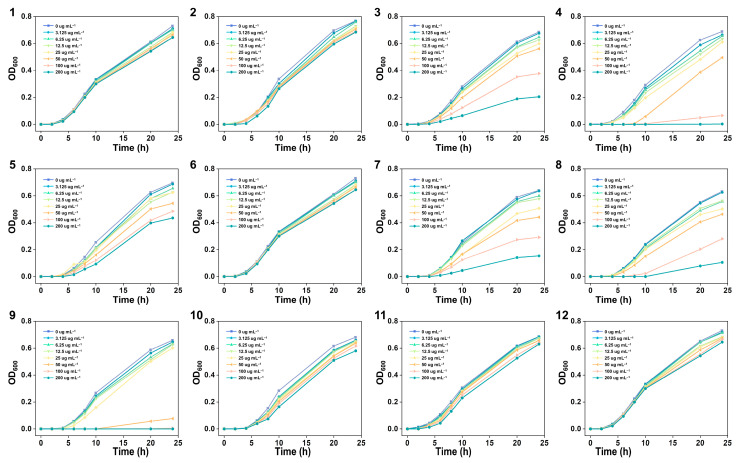
Bacterial growth curves of *S. aureus* after treatment with Schiff base ligands (**1** and **2**), metal complexes (**3**–**10**), and phosphane co-ligands (**11** and **12**).

First, the resazurin-based microtiter dilution assay (RMDA) method was used to evaluate the synergic antibacterial effect of Schiff bases (**1** and **2**), metal complexes (**3**–**10**), and phosphane co-ligands (**11** and **12**). Figure 4 and Figure 5 show the bacterial growth curves against Gram-negative (*E. coli*) and Gram-positive bacteria (*S. aureus*), respectively. The minimum inhibitory concentrations (MIC) were reported in Table 2 and half-maximal inhibitory concentrations (MIC_50_) values were calculated using computerized nonlinear regression analysis.

Generally, the growth of bacteria follows four phases: lag phase, logarithmic phase, stationary phase, and decline phase. When bacteria are exposed to external interferences or inhibitory factors, their growth may be significantly delayed or even completely suppressed, ultimately leading to growth cessation.

Based on the bactericidal growth curves, the free Schiff base ligands and phosphane co-ligands exhibited negligible toxicity against both Gram-negative and Gram-positive bacterial species, while all the metal complexes demonstrated different antibacterial activities. Especially, the silver complexes [Ag(HL^BSMe^)(PTA)]NO_3_ (**4**) and [Ag(HL^BSPh^)(PTA)]NO_3_ (**9**) showed a significant bactericidal effect. The MIC values of compound **4** against *E. coli* and *S. aureus* were 0.050 and 0.100 mg mL^−1^, respectively, while the MIC values of compound **9** were 0.025 and 0.050 mg mL^−1^, respectively. Interestingly, the MIC values of compound **4** were higher in comparison to the values of **9**; however, the bacterial growth curves indicated that the growth rate of bacteria treated with compound **4** was slower when the concentrations were below 0.025 mg mL^−1^. Their different bacterial growth behaviors can lie in the structure of the Schiff base ligands with the presence of a methyl or a phenyl substituent in compounds **4** and **9**, respectively. Previous studies have suggested that the bactericidal activity of silver compounds is primarily due to two distinct mechanisms, including the strong affinity to R-SH groups associated in amino acids or proteins and their ability to generate reactive oxygen species (ROS). Silver ions can readily bind to SH groups, such as those in cysteine residues, which can disrupt cellular metabolism and physiology by directly interfering with enzymatic functions or by destabilizing disulfide (S-S) bonds critical for maintaining protein structure. However, effective binding to SH groups requires silver ions to be sufficiently accessible and reactive [144,145].

**Figure 6:**
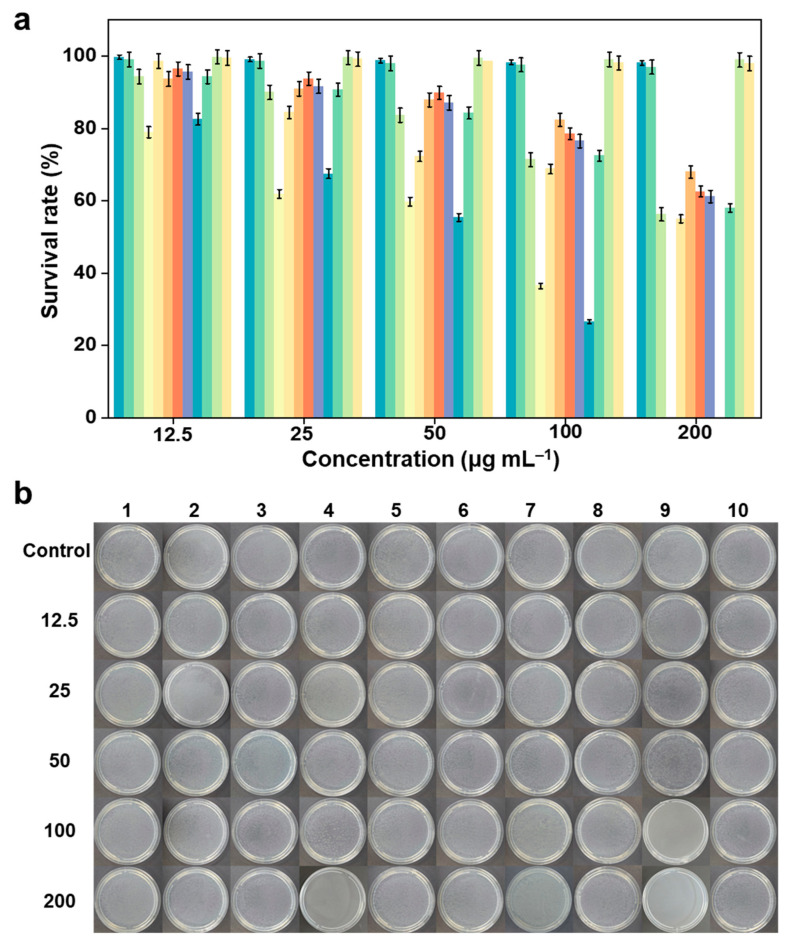
(**a**) The bacterial viability of compounds **1**–**12** (from left to right) with different concentration and (**b**) corresponding bacterial colony images of *E. coli*. Colonies were quantified with ImageJ software (1.50d) and presented as mean values from duplicate independent experiments. Error bars denote standard deviation. The significance level between the control and treated samples was established at *p* < 0.05.

**Figure 7:**
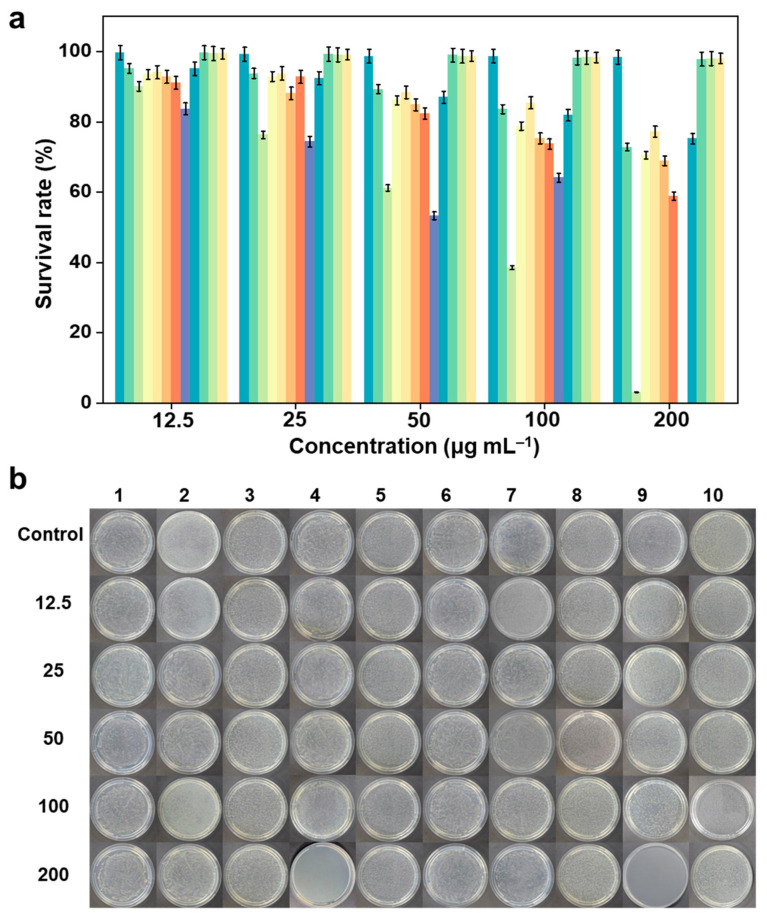
(**a**) The bacterial viability of compounds **1**–**12** (from left to right) with different concentrations and (**b**) corresponding bacterial colony images of *S. aureus*. Colonies were quantified with ImageJ software (1.50d) and presented as mean values from duplicate independent experiments. Error bars denote standard deviation. The significance level between the control and treated samples was established at *p* < 0.05.

The bactericidal activities of the compounds at different concentrations were further evaluated using the plate colony count method against *E. coli* (Figure 6) and *S. aureus* (Figure 7). The minimum bactericidal concentration (MBC) and half-maximal bactericidal concentration (MBC_50_) were calculated and included in Table 3.

The results demonstrate that the antimicrobial activities of the metal complexes **3**–**9** are significantly enhanced compared to the free Schiff base ligands, which can be explained by Tweedy’s chelation theory. Due to the electron donating properties of Schiff base ligands, a metal ion can share part of its charge through orbital overlap with Schiff base ligands, which reduces the polarity of the metal ion. Furthermore, this process increases the lipophilicity of the central metal atom, facilitating its penetration through the lipid layer of the microorganism, and blocking the interaction of enzymes on the cell membrane, thereby inhibiting the growth of the microorganism [146,147]. Subsequently, Cu(I) and Ag(I) coordination compounds were investigated to study the influence of the nature of a metal center in bioactivity. A comparison of the antimicrobial properties of compounds **3** and **4**, **6** and **8**, as well as **7** and **9** reveals that silver complexes exhibit superior antimicrobial effects against both *E. coli* and *S. aureus* strains compared to their copper-based counterparts. Among these metal complexes, compound **9** was found to be most active against the bacterial strains with MBC values of 0.100 and 0.200 mg mL^−1^ for *E. coli* and *S. aureus*, respectively. Additionally, compound **4** was prominently active with MBC values of 0.200 mg mL^−1^ against both tested bacterial strains. Compound **8** displayed moderate inhibitory effects with MBC_50_ values of 0.322 and 0.294 mg mL^−1^, however lower than the MBC_50_ values of 0.566 and 1.125 mg mL^−1^ for compound **6**. As previously mentioned, Ag(I) ions have the ability to alter the bacteria cell wall structure and cell constituent by irreversibly binding to cysteine residues, thereby disrupting essential enzyme systems and ultimately inhibiting bacterial proliferation [144,148].

Furthermore, a possible correlation between the biological activity of the compounds and their lipophilicity and hydrophilicity was discussed. By comparing compounds **6** and **7**, as well as **8** and **9**, it was observed that the antibacterial activity of the complexes with PTA as a co-ligand was superior to those with PPh_3_ as a co-ligand. Notably, the lipophilic triphenylphosphine and the hydrophilic 1,3,5-triaza-7-phosphaadamantane have been chosen as co-ligands because they can stabilize copper and silver in +1 oxidation state and can give also a different hydrophilic–lipophilic balance to the corresponding complexes. The antibacterial data indicate that the PTA co-ligands confer the related silver complexes the most appropriate balance between hydrophilicity and lipophilicity [149,150,151,152], what is in fact a major challenge in the development of antimicrobial agents. The presence of lipophilic groups enhances penetration ability to the bacterial lipophilic outer membrane which contains murein, thereby facilitating interactions with the bacterial replicative system [144,145]. However, several studies have also highlighted the importance of water solubility as a key parameter in antibacterial activity.

**Figure 8:**
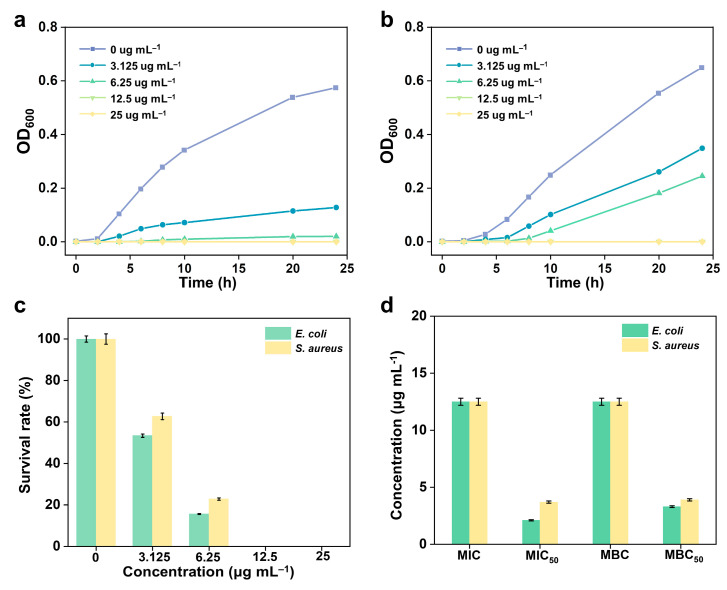
Bacterial growth curves of (**a**) *E. coli* and (**b**) *S. aureus* after treatment with ciprofloxacin. (**c**) Corresponding bacterial viability of *E. coli* (green) and *S. aureus* (yellow) after treatment with ciprofloxacin. (**d**) The summary of antimicrobial activity of ciprofloxacin.

The antibacterial performance of the synthesized compounds was systematically evaluated with respect to three critical determinants: (i) ligands chelation effects on bioavailability, (ii) properties of metal ions, and (iii) hydrophobic-lipophilic balance. Compared to the broad-spectrum antibiotic ciprofloxacin (Figure 8), although the synthesized metal complexes are less effective than the standard drug, compounds **4** and **9** have demonstrated significant potential as antibacterial agents. In addition, compared with other silver Schiff base metal complexes reported for antibacterial applications, the compounds **4** and **9** exhibits similar or lower MIC values [65,66,85,144,153,154,155,156].

## 3. Experimental Section

### 3.1. Materials and Instruments

All reagents were obtained from commercial suppliers and used as received. Melting Points (MP) were performed by an SMP3 Stuart Scientific Instrument (Bibby Sterilin Ltd., London, UK). Elemental analyses (C, H, N, S) (EA) were performed with a Fisons Instruments EA-1108 CHNS-O Elemental Analyzer (Thermo Fisher Scientific Inc., Waltham, MA, USA). Fourier-Transform InfraRed (FT-IR) spectra were recorded from 4000 to 700 cm^−1^ on a PerkinElmer Frontier Instrument (PerkinElmer Inc., Waltham, MA, USA), equipped with an Attenuated Total Reflection (ATR) unit using universal diamond top-plate as a sample holder. Abbreviations used in the analyses of the FT-IR spectra: br = broad, m = medium, mbr = medium broad, s = strong, sbr = strong broad, vs. = very strong, w = weak, wbr = weak broad. Nuclear Magnetic Resonance (NMR) spectra for the nuclei ^1^H, ^13^C and ^31^P were recorded with a Bruker 500 Ascend Spectrometer (Bruker BioSpin Corporation, Billerica, MA, USA; 500.13 MHz for ^1^H, 125.78 MHz for ^13^C, 202.46 MHz for ^31^P and 470.59 MHz for ^19^F). Tetramethylsilane (SiMe_4_) was used as external standard for the ^1^H- and ^13^C-NMR spectra, while 85% H_3_PO_4_ was used for the ^31^P-NMR spectra. The chemical shifts (δ) are reported in ppm, and coupling constants (J) are reported in hertz (Hz). Abbreviations used in the analyses of the NMR spectra: br = broad, d = doublet, dbr = broad doublet, m = multiplet, s = singlet, sbr = broad singlet, t = triplet. ElectroSpray Ionization Mass Spectra (ESI-MS) were recorded in positive- (ESI-MS(+)) or negative-ions (ESI-MS(−)) mode on a Waters Micromass ZQ Spectrometer equipped with a single quadrupole (Waters Corporation, Milford, MA, USA), using methanol or an acetonitrile mobile phase. The compounds were added to reagent grade methanol or acetonitrile to give approximately 0.1 mM solutions. These solutions were injected (1 µL) into the spectrometer fitted with an autosampler. The pump delivered the solutions to the mass spectrometer source at a flow rate of 200 μL/min and nitrogen was employed both as a drying and nebulizing gas. The capillary voltage was typically 2500 V. The temperature of the source was 100 °C, while the temperature of the desolvation was 400 °C. In the analyses of ESI-MS spectra, the confirmation of major peaks was supported by comparison of the observed and predicted isotope distribution patterns, the latter calculated using the IsoPro 3.1 computer software (T-Tech Inc., Norcross, GA, USA).

### 3.2. Synthesis

#### 3.2.1. Synthesis of the Ligand HL^BSMe^ (**1**)

The ligand (*E*)*-*2-(1-(benzylimino)ethyl)phenol*,* HL^BSMe^ (**1**) was prepared by the reaction of 1-(2-hydroxyphenyl)ethan-1-one (1.100 mmol, 0.150 g) and benzylamine (1.000 mmol, 0.107 g) in methanol (30 mL). The mixture was stirred at room temperature for 4 h, monitored via TLC, until the complete consumption of acylphenol. The mixture was evaporated at reduced pressure and dissolved in CH_2_Cl_2_ (25 mL) and 1M solution of NaOH (4 × 8 mL). After extraction, the organic phase was anhydrified with Na_2_SO_4_, evaporated and dried at reduced pressure, giving the product HL^BSMe^ in 80% yield. Solubility: CH_3_OH, CH_3_CH_2_OH, CH_3_CN, CHCl_3_, Acetone, EtOAc, DMSO. M.p.: 118–119 °C. FT-IR (cm^−1^): 3211wbr, 3108vw, 3061w, 3034w, 2918w, 2877w, 2814w (C-H); 1610s, 1572m (C=C and C=N); 1492m, 1451s, 1413m, 1376m, 1350m, 1330m, 1303s, 1263m, 1232m, 1181w, 1161m, 1129m, 1074w, 1055m, 1024m, 974m, 950m, 941m, 928m, 900m, 860m, 831m, 743vs, 733vs, 698vs, 639m, 584m, 559m, 525m, 501m. ^1^H-NMR (CDCl_3_, 293K): δ 2.51 (s, 3H, C*H*_3_), 4.87 (s, 2H, C*H*_2_), 6.83–7.61 (m, 9H, C*H*_ar_), 15.78 (sbr, 1H, O*H*). ^1^H-NMR (CD_3_CN, 293K): δ 2.48 (s, 3H, C*H*_3_), 4.83 (s, 2H, C*H*_2_), 6.83–7.71 (m, 9H, C*H*_ar_), 16.23 (sbr, 1H, O*H*). ^1^H-NMR (Acetone-d_6_, 293K): δ 2.52 (s, 3H, C*H*_3_), 4.88 (s, 2H, C*H*_2_), 6.80–7.72 (m, 9H, C*H*_ar_), 15.87 (sbr, 1H, O*H*). ^1^H-NMR (DMSO-d_6_, 293K): δ 2.51 (s, 3H, C*H*_3_), 4.83 (s, 2H, C*H*_2_), 6.78–7.72 (m, 9H, C*H*_ar_), 16.37 (sbr, 1H, O*H*). ^13^C{^1^H}-NMR (CDCl_3_, 293K): δ 14.7 (*C*H_3_), 53.4 (*C*H_2_), 117.2, 118.8, 119.5, 127.2, 127.5, 128.1, 128.8, 132.6, 138.5 (*C*H_Ar_), 163.8 (*C*OH), 172.2 (*C*=N). ESI-MS(+) (major positive ions, CH_3_CN), *m*/*z* (%): 226 (100) [L^BSMe^ + H]^+^. Elemental analysis (%) calculated for C_15_H_15_NO: C 79.97, H 6.71, N 6.22; found: C 80.25, H 6.54, N 6.36.

#### 3.2.2. Synthesis of the Ligand HL^BSPh^ (**2**)

The ligand (*E*)-2-((benzylimino)(phenyl)methyl)phenol, HL^BSPh^ (**2**) was prepared following the procedure described for ligand **1**, using (2-hydroxyphenyl)(phenyl)methanone (1.100 mmol, 0.218 g) and was obtained in 72% yield. Solubility: CH_3_OH, CH_3_CH_2_OH, CH_3_CN, CHCl_3_, Acetone, EtOAc, DMSO. M.p.: 89–91 °C. FT-IR (cm^−1^): 30,653w, 3026w, 2988w, 2966w, 2922w, 2858w (C-H); 1604s, 1563m (C=C and C=N); 1498m, 1446m, 1378m, 1355w, 1350w, 1299m, 1266m, 1227m, 1203m, 1165m, 1153m, 1126m, 1094m, 1075m, 1049m, 1028m, 993m, 948m, 912m, 856w, 829m, 798m, 752vs, 711m, 697s, 676m, 627m, 588m. ^1^H-NMR (CDCl_3_, 293K): δ 4.59 (s, 2H, C*H*_2_), 6.68–7.56 (m, 14H, C*H*_ar_), 15.64 (sbr, 1H, O*H*). ^1^H-NMR (CD_3_CN, 293K): δ 4.55 (s, 2H, C*H*_2_), 6.70–7.60 (m, 14H, C*H*_ar_), 15.42 (sbr, 1H, O*H*). ^13^C{^1^H}-NMR (CD_3_CN, 293K): δ 55.3 (*C*H_2_), 117.5, 117.6, 120.0, 127.1, 127.4, 127.6, 128.6, 128.8, 129.2, 131.6, 132.5, 133.9, 139.4 (*C*H_Ar_), 162.9 (*C*OH), 174.8 (*C*=N). ESI-MS(+) (major positive ions, CH_3_CN), *m*/*z* (%): 288 (100) [L^BSPh^ + H]^+^. Elemental analysis (%) calculated for C_20_H_17_NO: C 83.59, H 5.96, N 4.87; found: C 83.42, H 5.73, N 4.92.

#### 3.2.3. Synthesis of [Cu(HL^BSMe^)(PTA)_2_]PF_6_ (**3**)

Tetrakis(acetonitrile)copper(I)hexafluorophosphate (0.500 mmol, 0.186 g) and 1,3,5-triaza-7-phosphadaamantane (1.000 mmol, 0.157 g) were dissolved in CH_3_CN (30 mL) and the reaction was stirred for 2 h at room temperature. Then, the ligand HL^BSMe^ (0.500 mmol, 0.113 g) was added, and the reaction was stirred for 24 h at room temperature. The precipitate obtained was filtered and dried at reduced pressure giving the complex [Cu(HL^BSMe^)(PTA)_2_]PF_6_ in 66% yield. M.p.:191–193 °C. Solubility: CH_3_CN, DMSO. FT-IR (cm^−1^): 3200vbr (OH); 3062w, 3034w, 3268wbr, 2919wbr, 2879w (C-H); 1618m, 1574w (C=C and C=N); 1496w, 1449m, 1415w, 1376w, 1350w, 1294m, 1239m, 1161w, 1130w, 1107w, 1055w, 1040s, 969s, 949s, 895w; 831vs (PF_6_); 742vs, 733s, 698s, 639m, 583s, 556vs, 525m, 501m. ^1^H-NMR (CD_3_CN, 293K): δ 2.47 (s, 3H, C*H*_3_), 4.09 (s, 12H, NC*H*_2_P), 4.50–4.61 (AB q, 12H, NC*H*_2_N), 4.83 (s, 2H, C*H*_2_), 6.82–7.71 (m, 9H, C*H*_ar_), 16.25 (sbr, 1H, O*H*). ^31^P{^1^H}-NMR (CD_3_CN, 293K): δ −144.62 (septet, J_P−F_ = 706 Hz), −91.64 (sbr). ESI-MS(+) (major positive ions, CH_3_CN), *m*/*z* (%): 226 (100) [HL^BSMe^ + H]^+^, 287 (20) [Cu(HL^BSMe^)]^+^, 377 (10) [Cu(PTA)_2_]^+^, 445 (100) [Cu(HL^BSMe^)(PTA)]^+^. ESI-MS(−) (major negative ions, CH_3_CN), *m*/*z* (%): 145 (100) [PF_6_]^−^. Elemental analysis (%) calculated for C_27_H_39_CuF_6_N_7_OP_3_: C 43.35, H 5.25, N 13.11; found: C 44.14, H 5.39, N 13.05

#### 3.2.4. Synthesis of [Ag(HL^BSMe^)(PTA)]NO_3_ (**4**)

Silver nitrate (0.500 mmol, 0.085 g) and 1,3,5-triaza-phosphadaamantane (0.500 mmol, 0.078 g) were dissolved in CH_3_OH (30 mL) and the reaction was stirred for 2 h at room temperature. Afterwards, the ligand HL^BSMe^ (0.500 mmol, 0.113 g) was added, the reaction was stirred at room temperature for 24 h, then the solution was evaporated under reduced pressure. The product [Ag(HL^BSMe^)(PTA)]NO_3_ was obtained in 58% yield. M.p.: 206–208 °C. Solubility: CH_3_OH, CH_2_Cl_2_, CHCl_3_, CH_3_CN, DMSO. FT-IR (cm^−1^): 3210br (OH); 3060w, 3033w, 2917w, 2878w (C-H); 1616m, 1573w (C=C and C=N); 1494w; 1449m, 1425m, 1375m, 1334s, 1292s (NO_3_); 1240s, 1161m, 1129w, 1107m, 1074w, 1042w, 1013vs, 970vs, 947vs, 929m, 900m, 861w, 829w, 806s, 790sh, 743vs, 732vs, 698vs, 639m, 602s, 583s, 560s, 525m, 501m. ^1^H-NMR (DMSO-d_6_, 293K): δ 2.48 (s, 3H, C*H*_3_), 4.15 (s, 6H, NC*H*_2_P), 4.41–4.58 (AB q, 6H, NC*H*_2_N), 4.83 (s, 2H, C*H*_2_), 6.77–7.72 (m, 9H, C*H*_ar_), 16.38 (s, 1H, O*H*). ^31^P{^1^H}-NMR (DMSO-d_6_, 293K): δ −86.03 (s). ^31^P{^1^H}-NMR (CDCl_3_, 293K): δ −93.60 (s). ^31^P{^1^H}-NMR (CD_3_OD, 233K): δ −82.00 (sbr). ESI-MS(+) (major positive ions, CH_3_OH), *m*/*z* (%): 226 (100) [HL^BSMe^ + H]^+^, 248 (20) [HL^BSMe^ + Na]^+^, 423 (50) [Ag(PTA)_2_]^+^, 491 (40) [Ag(HL^BSMe^)(PTA)]^+^. ESI-MS(−) (major negative ions, CH_3_OH), *m*/*z* (%): 145 (100) [AgNO_3_ + NO_3_]^−^. Elemental Analysis (%) calculated for C_21_H_27_AgN_5_O_4_P: C 45.67, H 4.93, N 12.68; found: C 46.15, H 5.12, N 13.31.

#### 3.2.5. Synthesis of [Cu(L^BSMe^)_2_] (**5**)

Copper(II) acetate (0.250 mmol, 0.045 g) and the ligand HL^BSMe^ (0.500 mmol, 0.113 g) were solubilized in CH_3_OH (20 mL). The reaction was stirred at reflux for 4 h, giving a small amount of a dark green precipitate. After filtration, the mother liquors were dried at reduced pressure obtaining the green–blue solid [Cu(L^BSMe^)_2_] in 39% yield. M.p.:201–203 °C. Solubility: CH_3_OH, CH_3_CN, CH_2_Cl_2_, CHCl_3_, DMSO. FT-IR (cm^−1^): 3083w, 3058w, 3021w, 2974w, 2904w (C-H); 1597s, 1588s (C=C and C=N); 1536s; 1494m, 1469m, 1437s, 1366w, 1328s, 1262m, 1246m, 1215m, 1159m, 1132m, 1084w, 1055m, 1046m, 933m, 856m, 756s, 742s, 705s, 695s, 649m, 602m, 570m. ESI-MS(+) (major positive ions, CH_3_CN), *m*/*z* (%): 226 (100) [HL^BSMe^ + H]^+^, 287 (40) [Cu(L^BSMe^)]^+^, 329 (30) [Cu(L^BSMe^) + CH_3_CN]^+^, 511 (90) [Cu(L^BSMe^)_2_ + H]^+^, 576 (80) [Cu(L^BSMe^)_2_ + Na + CH_3_CN]^+^. ESI-MS(−) (major negative ions, CH_3_CN), *m*/*z* (%): 240 (100) [L^BSMe^]^−^. Elemental analysis (%) calculated for C_30_H_28_CuN_2_O_2_: C 70.36, H 5.51, N 5.47; found: C 69.79, H 5.41, N 5.25.

#### 3.2.6. Synthesis of [Cu(HL^BSPh^)(PPh_3_)_2_]PF_6_·2CH_3_CN (**6**)

Tetrakis(acetonitrile)copper(I)hexafluorophosphate (0.500 mmol, 0.186 g) and triphenylphosphine (1.000 mmol, 0.262 g) were dissolved in CH_3_CN (30 mL) and the reaction was stirred for 3 h at room temperature. The ligand HL^BSPh^ (0.500 mmol, 0.144 g) was added, and the reaction was stirred for 24 h at room temperature. The solvent was removed at reduced pressure and the solid obtained was washed with Et_2_O. A precipitate was recovered by filtration giving the complex [Cu(HL^BSPh^)_2_(PPh_3_)]PF_6_·2CH_3_CN in 83% yield. M.p.:161–164 °C Solubility: CH_3_OH, CH_3_CN, CHCl_3_, CH_2_Cl_2_, DMSO. FT-IR (cm^−1^): 3070w, 3050w, 3028w, 2938w, 2916w (C-H); 2301wbr, 2271wbr (CH_3_CN); 1605m, 1572w (C=C and C=N); 1497w, 1480m, 1450w, 1434s, 1369w, 1346w, 1328w, 1304m, 1258m, 1245w, 1181w, 1150w, 1112w, 1095m, 1072w, 1047w, 1035w, 999w, 972w, 923w, 879w, 859msh; 832vs (PF_6_); 775m, 766m, 744vs, 716m, 694vs, 646m, 618w, 557vs, 541m, 527m, 516s, 504vs. ^1^H-NMR (CD_3_CN 293K): δ 1.99 (s, 6H, C*H*_3_CN), 4.55 (s, 2H, C*H*_2_), 6.71–7.69 (m, 44H, C*H*_ar_), 15.41 (s, 1H, O*H*). ^1^H-NMR (CDCl_3_ 293K): δ 2.08 (s, 6H, C*H*_3_CN), 4.61 (s, 2H, C*H*_2_), 6.70–7.71 (m, 44H, C*H*_ar_). ^31^P{^1^H}-NMR (CD_3_CN, 293K): δ −144.62 (septet, J_P−F_ = 706 Hz), −0.30 (sbr). ^31^P{^1^H}-NMR (CDCl_3_, 293K): δ −144.25 (septet, J_P−F_ = 713 Hz), −0.61 (s). ESI-MS(+) (major positive ions, CH_3_CN), *m*/*z* (%): 366 (100) [Cu(PPh_3_) + CH_3_CN]^+^, 589 (100) [Cu(PPh_3_)_2_]^+^. ESI-MS(−) (major negative ions, CH_3_CN), *m*/*z* (%): 145 (100) [PF_6_]^−^. Elemental Analysis (%) calculated for C_60_H_53_CuF_6_N_3_OP_3_: C 65.36, H 4.85, N 3.81; found: C 65.25, H 4.88, N 3.66.

#### 3.2.7. Synthesis of [Cu(HL^BSPh^)(PTA)_2_]PF_6_·2H_2_O (**7**)

Tetrakis(acetonitrile)copper(I)hexafluorophosphate (0.500 mmol, 0.186 g) and 1,3,5-triaza-7-phosphadaamantane (1.000 mmol, 0.157 g) were dissolved in CH_3_CN (30 mL) and the reaction was stirred for 2 h at room temperature. The ligand HL^BSPh^ (0.500 mmol, 0.144 g) was added giving a yellow mixture. After 24 h at room temperature, the solvent was evaporated under reduced pressure obtaining the complex [Cu(HL^BSPh^)(PTA)_2_]PF_6_·2H_2_O in 72% yield. M.p.: 225–228 °C. Solubility: CH_3_OH, CH_3_CH_2_OH, CH_2_Cl_2_, CHCl_3_, CH_3_CN, DMSO. FT-IR (cm^−1^): 3345wbr, 3188wbr (OH); 3063wbr, 3028w, 2916w, 2878w (C-H); 1605s, 1571m (C=C and C=N); 1497m, 1441m, 1419m; C=N); 1346w, 1328w, 1303m, 1257m, 1242s, 1171w, 1150m, 1111m, 1081w, 1045w, 1015s, 970s, 949s, 923m, 876w; 832vs (PF_6_); 775s, 765s, 751vs, 715vs, 698vs, 646s, 611m, 582s, 555vs, 506s. ^1^H-NMR (CD_3_CN, 293K): δ 2.24 (s, 4H, *H*_2_O), 4.08 (s, 12H, NC*H*_2_N), 4.50–4.60 (AB q, 14H, NC*H*_2_P and C*H*_2_), 6.70–7.62 (m, 14H, C*H*_ar_), 15.45 (s, 1H, O*H*). ^31^P{^1^H}-NMR (CD_3_CN, 293K): δ −144.58 (septet, J_P−F_ = 706 Hz), −89.66 (br). ESI-MS(+) (major positive ions, CH_3_CN), *m*/*z* (%): 261 (90) [Cu(PTA) + CH_3_CN]^+^, 288 (20) [HL^BSPh^ + H]^+^, 377 (10) [Cu(PTA)_2_]^+^, 507 (15) [Cu(HL^BSPh^)(PTA)]^+^. ESI-MS(−) (major negative ions, CH_3_CN), *m*/*z* (%): 145 (100) [PF_6_]^−^. Elemental Analysis (%) calculated for C_32_H_45_CuF_6_N_7_O_3_P_3_: C 45.42, H 5.36, N 11.59; found: C 44.69, H 5.03, N 12.24.

#### 3.2.8. Synthesis of [Ag(HL^BSPh^)(PPh_3_)_2_]NO_3_ (**8**)

Silver nitrate (0.500 mmol, 0.085 g) and triphenylphosphine (1.000 mmol, 0.262 g) were dissolved in CH_3_CN (30 mL) and the reaction was stirred for 2 h at room temperature. Afterwards, the ligand HL^BSPh^ (0.500 mmol, 0.144 g) was added, and the reaction was stirred at room temperature for 24 h. The precipitate obtained was filtered and dried under reduced pressure giving the product [Ag(HL^BSPh^)(PPh_3_)_2_]NO_3_ in 40% yield. M.p.: 187–189 °C. Solubility: CH_3_OH, CH_2_Cl_2_, CHCl_3_, CH_3_CN. FT-IR (cm^−1^): 3059wbr, 3028w, 2918w (C-H); 1605m, 1571w (C=C and C=N); 1497w, 1489w; 1479m, 1436m, 1397s, 1346w, 1328w, 1296s (NO_3_); 1257m, 1179w, 1150m, 1112w, 1095m, 1072w, 1047m, 1025m, 997m, 971w, 918s, 864w, 849w, 833w, 825w, 818w, 775m, 766m, 751vs, 742vs, 715s, 693vs, 646m, 618w, 567w, 547w, 513ssh, 503vs. ^1^H-NMR (CDCl_3_, 293K): δ 4.58 (s, 2H, C*H*_2_), 6.68–7.70 (m, 44H, C*H*_ar_), 15.67 (s, 1H, O*H*). ^1^H-NMR (DMSO-d_6_, 293K): δ 4.52 (s, 2H, C*H*_2_), 6.71–7.63 (m, 44H, C*H*_ar_), 15.46 (s, 1H, O*H*). ^31^P{^1^H}-NMR (CDCl_3_, 293K): δ 14.68 (sbr). ^31^P{^1^H}-NMR (CD_3_CN, 233K): δ 8.69 (d, *J*(Ag-^31^P) = 478 Hz). ESI-MS(+) (major positive ions, CH_3_CN), *m*/*z* (%): 288 (10) [HL^BSPh^ + H]^+^, 371 (30) [Ag(PPh_3_)]^+^, 410 (30) [Ag(PPh_3_) + CH_3_CN]^+^, 630 (100) [Ag(PPh_3_)_2_]^+^. ESI-MS(−) (major negative ions, CH_3_OH), *m*/*z* (%): 231 (100) [AgNO_3_ + NO_3_]^−^. Elemental analysis (%) calculated for C_56_H_47_AgN_2_O_4_P_2_: C 68.51, H 4.83, N 2.85; found: C 69.43, H 4.87, N 2.90.

#### 3.2.9. Synthesis of [Ag(HL^BSPh^)(PTA)]NO_3_ (**9**)

Silver nitrate (0.500 mmol, 0.085 g) and 1,3,5-triaza-7-phosphadaamantane (0.500 mmol, 0.078 g) were dissolved in CH_3_CN. After 2 h, the ligand HL^BSPh^ (0.500 mmol, 0.145 g) was added and the reaction was stirred at room temperature for 22 h and refluxed for further 4 h. Successively, the solvent was removed under reduced pressure and the yellow solid product [Ag(HL^BSPh^)(PTA)]NO_3_·was obtained in 55% yield. M.p.: 232–234 °C. Solubility: CH_3_OH, CH_3_CN, CHCl_3_, CH_2_Cl_2_, DMSO. FT-IR (cm^−1^): 3200wbr (OH); 3064wbr, 3029w, 2916w (C-H); 1605s, 1571m (C=C and C=N); 1516w, 1497w, 1489w; 1450m, 1441m, 1425m, 1416m, 1374m, 1329s, 1294s (NO_3_); 1257s, 1241s, 1170w, 1150m, 1110m, 1081w, 1072w, 1045m, 1013s, 970vs, 947vs, 919s, 865w, 851m, 825m, 775s, 766s, 751vs, 715vs, 698vs, 646s, 605s, 582s, 564s, 547m, 506s. ^1^H-NMR (DMSO-d_6_, 293K): δ 4.16 (s, 6H, NC*H*_2_N), 4.42–4.58 (dd, 6H, NC*H*_2_P and C*H*_2_), 6.71–7.63 (m, 14H, C*H*_ar_), 15.45 (sbr, 1H, O*H*). ^31^P{^1^H}-NMR (CDCl_3_, 293K): δ −94.43 (s). ^31^P{^1^H}-NMR (CD_3_CN, 233K): δ −86.09 (sbr). ESI-MS(+) (major positive ions, CH_3_CN), *m*/*z* (%): 158 (90) [PTA + H]^+^, 260 (80) [Ag(PTA)]^+^, 288 (100) [HL^BSPh^ + H]^+^, 423 (50) [Ag(PTA)_2_]^+^, 553 (10) [Ag(HL^BSPh^)(PTA)]^+^. ESI-MS(−) (major negative ions, CH_3_CN), *m*/*z* (%): 231 (70) [AgNO_3_ + NO_3_]^−^. Elemental analysis (%) calculated for C_26_H_27_AgN_5_O_4_P: C 50.83, H 4.76, N 11.50; found: C 51.27, H 5.12, N 11.91.

#### 3.2.10. Synthesis of [Cu(L^BSPh^)_2_] (**10**)

Copper(II) acetate (1.000 mmol, 0.200 g) was dissolved in CH_3_OH obtaining an intense blue solution. At the same time, the ligand HL^BSPh^ (2.000 mmol, 0.115 g) was solubilized in CH_3_CN. The two solutions were mixed, and it was stirred at room temperature for 1 h and then refluxed for 23 h. After 24 h, the dark green precipitate formed was filtered and dried under reduced pressure giving the complex [Cu(L^BSPh^)_2_] in 77% yield. A batch of good quality crystals of [Cu(L^BSPh^)_2_], suitable for X-ray analysis, was obtained by slow evaporation of a toluene solution of **10**. M.p.: 228–230 °C. Solubility: CH_3_OH, CH_3_CH_2_OH, CH_2_Cl_2_, CH_3_Cl, EtOAc, CH_3_CN, DMSO, Acetone. FT-IR (cm^−1^): 3080w, 3044w, 3025w, 2940w (C-H); 1599s, 1568s (C=C and C=N); 1529vs; 1491m, 1456m, 1440vs, 1341vs, 1257s, 1241s, 1203w, 1178w, 1142vs, 1120m, 1075m, 1051m, 1039w, 1024m, 998w, 961m, 935s, 914m, 847s, 777m, 765m, 747vs, 720s, 702vs, 691vs, 641w, 629m, 618m. ESI-MS(+) (major positive ions, CH_3_CN), *m*/*z* (%): 288 (100) [HL^BSPh^ + H]^+^, 349 (70) [Cu(L^BSPh^)]^+^, 390 (100) [Cu(L^BSPh^) + CH_3_CN]^+^, 636 (20) [2HL^BSPh^ + H]^+^, 658 (20) [2HL^BSPh^ + Na]^+^, 741 (25) [2HL^BSPh^ + Na + 2CH_3_CN]^+^. Elemental analysis (%) calculated for C_40_H_32_CuN_2_O_5_: C 75.51, H 5.07, N 4.40; found: C 75.38, H 5.12, N 4.41.

### 3.3. Crystallographic Data Collection and Refinement

A batch of very good crystals were obtained by slow recrystallization of the [Cu(L^BSPh^)_2_] complex from a toluene solution. The specimens were of prismatic shape and intensely colored in green. The selected item was fixed to a glass capillary with Loctite glue and mounted on the head of a four-circle, kappa-geometry single-crystal OD Rigaku diffractometer. The data collection was performed under a graphite-monochromated Cu Kα radiation (*λ* = 1.54184 Å) at room temperature [295.1(3) K], and reflections were collected with the *ω*–scans technique. The preliminary screening of the sample allowed us to plan the data collection in the 1024 × 1024 pixel mode and 2 × 2 pixel binning. Intensity spots were recorded with an Atlas CCD area detector recently obtained by Prof. K Rissanen (University of Jyväskylä, Finland) in replacement of the former EOS camera. The crude diffraction data were collected working with the CrysAlisPro software, Version 1.171.43.141a [157], and treated for Lorentz and polarization effects. An empirical correction for absorption based on a multi-scan approach using equivalent reflections was applied by means of the SCALE3 ABSPACK algorithm, also accessible via the CrysAlisPro software. Two reference frames were collected every 50 frames to ensure crystal and equipment stability, showing no sign of sample deterioration or motion.

The complex crystallizes in the monoclinic system and the structure was solved in the *I*2/*a* space group; the unit cell parameters have been refined by working on the least-squares refinement of 18,133 strong reflections gathered during the whole experiment. The structure solution was performed by direct phasing and refined by full-matrix least-squares methods based on *F*_o_^2^ through the OLEX2 program interface [158] with the SHELXT and SHELXL [159,160] programs. The asymmetric unit contains half of a molecule, with the copper ion lying on a proper two-fold axis, and the whole cell hosts four units of the complex, which are very efficiently packed (no solvent accessible voids). In the final stages of the refinement, non-H atoms were allowed to vibrate anisotropically, whereas the H atoms were placed in calculated positions and refined as a riding model, with their displacement parameters calculated as 1.2 times the *U*_eq_ of the ‘parent’ atom.

At this level, it turned out that the phenyl ring (C15/C20) belonging to the benzylimino moiety of the ligand can assume two possible arrangements (involved atoms labelled with a final ‘A’). The sum of the pertinent occupation factors for each disordered position were constrained to unity, with finally refined sofs of 0.57/0.43 for untagged and tagged atoms, respectively. A regular hexagon geometry with bond distances of 1.3900 Å was imposed on the disordered positions of the phenyl ring via the AFIX 6 constraint to ensure refinement convergence and to prevent some atoms from assuming non-positive definite displacement parameters. A summary of crystal and data collection parameters is given below in Table 4; full listings of bond lengths and angles are in Tables S2 and S3. Representations of the asymmetric unit of [Cu(L^BSPh^)_2_] and of the entire complex (Figure 1 and Figure 2) with and without the disordered rings, highlighting the selected numbering scheme, have been prepared with the Mercury 4.2.0 software [161]. Complete listings of atomic coordinates, bond lengths, bond angles, and anisotropic thermal parameters are available as supporting information, in the form of the .cif file, that has been deposited at the Cambridge Crystallographic Data Center (CCDC) with deposition number 2433689. These data can be obtained free of charge via www.ccdc.cam.ac.uk/structures (accessed on 21 April 2025).

### 3.4. Antibacterial Screening

The synthesized Schiff bases HL^BSMe^ (**1**) and HL^BSPh^ (**2**), the related metal complexes **3**–**10**, and the free phosphane co-ligands triphenylphosphine (**11**) and, 3,5-triaza-7-phosphadaamantane (**12**), were evaluated for their in-vitro antibacterial activity against Gram-negative (*Escherichia coli* China-bio-00021) and Gram-positive bacteria (*Staphylococcus aureus* ATCC 25923), with ciprofloxacin serving as standard antibacterial agent. The antimicrobial properties of the compounds were evaluated using the standard broth microdilution method in 96-well cell culture plates following the reported method [162]. Firstly, the compounds were dissolved in PBS buffer containing 1% DMSO solution and stirred for 20 min until forming the homogeneous suspension (1.0 mg mL^−1^). Subsequently, a series of compounds solutions (50 µL) were prepared in a 96-well plate using the double dilution method, followed by the addition of bacterial suspensions. The final compound concentrations were 3.125, 6.25, 12.5, 25, 50, 100, and 200 μg mL^−1^, and the final bacterial concentration was 5 × 10^6^ CFU mL^−1^. The group without an antimicrobial agent was set as the control. Then, the 96-well plate was incubated at 37 °C for 24 h and the optical density (OD_600_) values of each well was recorded at different times (0, 2, 4, 6, 8, 10, 12, 20, and 24 h) using a microplate reader (Thermo Scientific, Waltham, MA, USA). According to the principle that the turbidity of the culture increases with the proliferation of bacteria, the growth state of bacteria can be evaluated by detecting the absorbance value of the bacterial suspension at 600 nm. In addition, the synthesized compounds were evaluated for their in-vitro antibacterial activity against two bacterial strains using the plate count method. Typically, 100 µL of bacterial suspension (10^6^ CFU mL^−1^) was mixed with 100 µL of compound solution at different concentrations (25, 50, 100, 200 and 400 μg mL^−1^). Subsequently, 100 µL of the mixed solution was spread on LB agar plates. After incubation at 37 °C for another 24 h, the surviving bacteria on each plate were counted. The antibacterial ratio was calculated using the following formula, where C*_b_* and C*_e_* are the numbers of colonies in the blank group and the experimental group, respectively, as follows:

$$\mathrm{Antibacterial}\;\mathrm{efficiency}\;=\;\frac{C_{b}-C_{e}}{C_{b}}\times 100\%$$

#### Statistical Analysis

The collected data were analyzed using GraphPad Prism 10 or OriginPro 2021 (9.8) software. Statistical significance was defined as *p* < 0.05, and the results were presented to three decimal places.

## 4. Conclusions

In this study, we report the synthesis, characterization, and antibacterial evaluation of Cu(I) and Ag(I) complexes supported by two phenoxy-ketimines ligands. In particular, 2-(1-(benzylimino)ethyl)phenol (HL^BSMe^) and 2-((benzylimino)(phenyl)methyl)phenol (HL^BSPh^), characterized by the presence of methyl or phenyl substituents on the imine group, were employed for the preparation of the complexes **2**–**4** and **6**–**9**, using the lipophilic PPh_3_ and the hydrophilic PTA as co-ligands to stabilize the metal in +1 oxidation state. The analogous copper(II) complexes **5** and **10** were synthesized using the copper(II) acetate salt Cu(CH_3_COO)_2_ and the ligands HL^BSMe^ and HL^BSPh^, respectively. All species were fully characterized both in the solid state and in solution. The single-crystal XRD allowed us to describe the molecular structure of **10**, the first reported complex of Cu(II) with the HL^BSPh^ ligand, where the copper ion has a slightly distorted square planar environment, as well as to highlight the efficient crystal packing, even in the absence of strong canonical hydrogen bonds, largely driven by π····π and C-H····π nonbonding interactions. The antimicrobial properties of the new copper and silver complexes as well as of the corresponding uncoordinated ligands were evaluated against *E. coli* and *S. aureus*. The results revealed that all the complexes exhibited moderate to excellent antibacterial efficacy. Among them, the Ag(I) complexes displayed the highest antibacterial activity. Specifically, [Ag(HL^BSPh^)(PTA)]NO_3_ demonstrated the most promising antibacterial performance, with MIC values of 0.025 and 0.05 mg mL^−1^ against *E. coli* and *S. aureus*, respectively, while the corresponding MBC values were 0.100 and 0.200 mg mL^−1^.

This study provides new insights into the design of antimicrobial agents, highlighting the potential for rationally designing and synthesizing novel, highly efficient antibacterial compounds. To further enhance the antibacterial activity of the silver complexes of phenoxy-ketimine Schiff base ligands, future research should focus on optimizing the synthesis of the most promising candidates, including fine-tuning the electronic and steric effects of both primary and auxiliary ligands, as well as modifying the solubility properties of the related complexes. In addition, detailed mechanistic studies could reveal specific pathways and interactions, facilitating the design of more effective compounds. Future investigations should also prioritize an in-depth exploration of structure-activity relationships to elucidate the role of distinctive phosphanes and of different substituents on the phenoxy-ketimines ligands in biological activity.

## Figures and Tables

**Table 1:** Relevant nonbonding interactions for [Cu(L^BSPh^)_2_] (**10**).

A Atom	D Atom	P Atom ^a^	A····D (Å)	A····D–P (°)	Symmetry Op. ^b^
O1	H18	C18	2.81	171.8	3/2 − *x*, *y*, 1 − *z*
O1	H18A	C18	2.85	159.9	3/2 − *x*, *y*, 1 − *z*
C15/C20	H11	C11 ^c^	2.78 ^d^	168.8 ^d^	1 − *x*, −1/2 + *y*, 1/2 − *z*
C15A/C20A	H11	C11 ^c^	2.78 ^d^	165.0 ^d^	1 − *x*, −1/2 + *y*, 1/2 − *z*
C1	H12	C12 ^c^	2.91	147.7	1 − *x*, 1 − *y*, 1 − *z*
C2	H12	C12 ^c^	2.91	145.7	1 − *x*, 1 − *y*, 1 − *z*
C15/C20	C15/C20 ^e^		4.20 ^f^	26.6 ^g^	1/2 − *x*, *y*, 1 − *z*
C15A/C20A	C15A/C20A ^e^		4.03 ^f^	23.4 ^g^	1/2 − *x*, *y*, 1 − *z*

^a^ Atom to which D atom is bound; ^b^ symmetry operations related to asymmetric unit = *x*, *y*, *z*; ^c^ C-H····π interactions; ^d^ distances and angles referring to selected ring centroids; ^e^ π····π interactions; ^f^ distance between ring centroids; ^g^ angle between mean planes encompassing phenyl rings.

**Table 2:** MIC and MIC_50_ values of Schiff base ligands (**1** and **2**), metal complexes (**3**–**10**), and co-ligands (**11** and **12**) in-vitro antimicrobial screening.

		*E. coli*		*S. aureus*	
N.	Tested Compounds	MIC	MIC_50_	MIC	MIC_50_
**1**	HL^BSMe^	/	/	/	/
**2**	HL^BSPh^	/	/	/	/
**3**	[Cu(HL^BSMe^)(PTA)_2_]PF_6_	/	0.057 (0.076)	/	0.116 (0.149)
**4**	[Ag(HL^BSMe^)(PTA)]NO_3_	0.050 (0.090)	0.012 (0.021)	0.100 (0.181)	0.061 (0.110)
**5**	[Cu(L^BSMe^)_2_]	/	0.110 (0.210)	/	0.381 (0.738)
**6**	[Cu(HL^BSPh^)(PPh_3_)_2_]PF_6_·2CH_3_CN	/	/	/	/
**7**	[Cu(HL^BSPh^)(PTA)_2_]PF_6_·2H_2_O	/	0.114 (0.130)	/	0.084 (0.100)
**8**	[Ag(HL^BSPh^)(PPh_3_)_2_]NO_3_	/	0.098 (0.100)	/	0.080 (0.079)
**9**	[Ag(HL^BSPh^)(PTA)]NO_3_	0.025 (0.040)	0.010 (0.016)	0.050 (0.081)	0.036 (0.058)
**10**	[Cu(L^BSPh^)_2_]	/	/	/	/
**11**	PPh_3_	/	/	/	/
**12**	PTA	/	/	/	/

MIC and MIC_50_ calculated in mg mL^−1^ (mM in parentheses); “/”: more than 0.381 mg mL^−1^ and 0.738 mM.

**Table 3:** MBC and MBC_50_ values of Schiff base ligands (**1** and **2**), metal complexes (**3**–**10**), and co-ligands (**11** and **12**) in-vitro antimicrobial screening.

		*E. coli*		*S. aureus*	
N.	Tested Compounds	MBC	MBC_50_	MBC	MBC_50_
**1**	HL^BSMe^	/	/	/	/
**2**	HL^BSPh^	/	/	/	/
**3**	[Cu(HL^BSMe^)(PTA)_2_]PF_6_	/	0.262 (0.350)	/	0.642 (0.858)
**4**	[Ag(HL^BSMe^)(PTA)]NO_3_	0.200 (0.362)	0.048 (0.086)	0.200 (0.362)	0.062 (0.112)
**5**	[Cu(L^BSMe^)_2_]	/	0.243 (0.474)	/	0.578 (1.128)
**6**	[Cu(HL^BSPh^)(PPh_3_)_2_]PF_6_·2CH_3_CN	/	0.566 (0.513)	/	1.125 (1.020)
**7**	[Cu(HL^BSPh^)(PTA)_2_]PF_6_·2H_2_O	/	0.319 (0.377)	/	0.551 (0.651)
**8**	[Ag(HL^BSPh^)(PPh_3_)_2_]NO_3_	/	0.322 (0.327)	/	0.294 (0.299)
**9**	[Ag(HL^BSPh^)(PTA)]NO_3_	0.100 (0.163)	0.046 (0.075)	0.200 (0.326)	0.068 (0.110)
**10**	[Cu(L^BSPh^)_2_]	/	0.282 (0.443)	/	0.875 (1.375)
**11**	PPh_3_	/	/	/	/
**12**	PTA	/	/	/	/

MBC and MBC_50_ calculated in mg mL^−1^ (mM in parentheses); “/”: more than 1.125 mg mL^−1^ and 1.375 mM.

**Table 4:** Summary of crystal and data collection parameters for [Cu(L^BSPh^)_2_] (**10**).

Empirical Formula	C_40_H_32_CuN_2_O_2_
Formula weight	636.21
Temperature/K	295.1(3)
Radiation	Cu Kα (*λ* = 1.54184)
Crystal system	monoclinic
Space group	*I*2/*a*
*a*/Å	20.4267(4)
*b*/Å	8.7751(2)
*c*/Å	18.3240(3)
*α*/°	90
*β*/°	101.135(2)
*γ*/°	90
Volume/Å^3^	3222.68(11)
*Z*	4
*ρ*_calc_ g/cm^3^	1.311
*μ*/mm^−1^	1.246
*F* (000)	1324.0
Crystal size/mm^3^	0.42 × 0.4 × 0.2
2*θ* range for data collection/°	8.824 to 155.422
Index ranges	−20 ≤ *h* ≤ 25, −11 ≤ *k* ≤ 11, −23 ≤ *l* ≤ 23
Reflections collected	36,327
Independent reflections	3416 [*R*_int_ = 0.0377]
Data/restraints/parameters	3416/0/247
Goodness-of-fit on *F*^2^	1.034
Final *R* indexes [*I* ≥ 2σ (*I*)]	*R*_1_ = 0.0331, *wR*_2_ = 0.0927
Largest diff. peak/hole/e Å^−3^	0.17/−0.31

Goodness–of–fit = [Σ (*w* (*F*_o_^2^ − *F*_c_^2^)^2^]/(*N*_obsvns_ − *N*_params_)]^1/2^, based on all data; *R*_1_ = Σ ||*F*_o_| − |*F*_c_||/Σ |*F*_o_|; *wR*_2_ = [Σ *w* (*F*_o_^2^ − *F*_c_^2^)^2^/Σ *w* (*F*_o_^2^)^2^]^1/2^.

## Data Availability

The data presented in this study are available on request from the corresponding authors. CCDC 2433689 contains the supplementary crystallographic data for this paper, available free of charge from the Cambridge Crystallographic Data Centre via www.ccdc.cam.ac.uk/structures (accessed on 21 April 2025).

## Supplementary Materials

The following supporting information can be downloaded at: https://www.mdpi.com/article/10.3390/molecules30091893/s1, Figures S1–S35: FT-IR, ^1^H-, ^13^C-, and ^31^P-NMR spectra of compounds **1**–**10**; Figures S36–S38: packing diagrams highlighting nonbonding interaction network for complex **10**; Table S1: ^1^H- and ^31^P{^1^H}-NMR selected data; Tables S2 and S3: full listings of bond lengths and angles of the complex **10**.
