# Supplementary material for: New Cu(II), Cu(I) and Ag(I) Complexes of Phenoxy-Ketimine Schiff Base Ligands: Synthesis, Structures and Antibacterial Activity
Source: Molecules. 2025 Apr 24;30(9):1893. doi: 10.3390/molecules30091893 (PMC12073749; doi:10.3390/molecules30091893)
Supplement: Supplementary file 1 [file molecules-30-01893-s001.zip › molecules-3596822-supplementary.pdf]

SUPPORTING INFORMATION

New Cu(II), Cu(I) and Ag(I) Complexes of Phenoxy-Ketimine Schiff Base Ligands: Synthesis, Structures and Antibacterial Activity

1 School of Science and Technology, Chemistry Division, University of Camerino, Via Madonna Delle Carceri (ChIP), 62032 Camerino, Italy; miriam.caviglia@unicam.it (M.C.); zhenzhen.li@unicam.it (Z.L.); carlo.santini@unicam.it (C.S.); jo.delgobbo@unicam.it (J.D.G.); cristina.cimarelli@unicam.it (C.C.)
2 College of Material and Chemical Engineering, Institute of New Energy Science and Technology, Zhengzhou University of Light Industry, Zhengzhou 450001, China
3 Department of Pharmaceutical and Pharmacological Sciences, University of Padova, Via Marzolo 5, 35131 Padova, Italy
* Correspondence: dumiao@zzuli.edu.cn (M.D.); alessandro.dolmella@unipd.it (A.D.); maura.pellei@unicam.it (M.P.)

Table of contents

Figure S1. FT-IR spectrum of HL^{BSMe} (**1**).

Figure S2. ^{1}H-NMR spectrum of HL^{BSMe} (**1**) in $CDCl_3$.

Figure S3. ^{1}H-NMR spectrum of HL^{BSMe} (**1**) in CD_3CN.

Figure S4. $^{13}C\{^{1}H\}$-NMR spectrum of HL^{BSMe} (**1**) in $CDCl_3$.

Figure S5. FT-IR spectrum of HL^{BSPh} (**2**).

Figure S6. ^{1}H-NMR spectrum of HL^{BSPh} (**2**) in $CDCl_3$.

Figure S7. ^{1}H-NMR spectrum of HL^{BSPh} (**2**) in CD_3CN.

Figure S8. $^{13}C\{^{1}H\}$-NMR spectrum of HL^{BSPh} (**2**) in CD_3CN.

Figure S9. FT-IR spectrum of $[Cu(HL^{BSMe})(PTA)_2]PF_6$ (**3**).

Figure S10. ^{1}H-NMR spectrum of $[Cu(HL^{BSMe})(PTA)_2]PF_6$ (**3**) in CD_3CN.

Figure S11. $^{31}P\{^{1}H\}$-NMR spectrum of $[Cu(HL^{BSMe})(PTA)_2]PF_6$ (**3**) in CD_3CN.

Figure S12. FT-IR spectrum of $[Ag(HL^{BSMe})(PTA)]NO_3$ (**4**).

Figure S13. ^{1}H-NMR spectrum of [Ag(HL^{BSMe})(PTA)]NO_3 (**4**) in DMSO-d_6.

Figure S14. ^{31}P{^{1}H}-NMR spectrum of [Ag(HL^{BSMe})(PTA)]NO_3 (**4**) in $CDCl_3$.

Figure S15. ^{31}P{^{1}H}-NMR spectrum of [Ag(HL^{BSMe})(PTA)]NO_3 (**4**) in DMSO-d_6.

Figure S16. ^{31}P{^{1}H}-NMR spectrum of [Ag(HL^{BSMe})(PTA)]NO_3 (**4**) in CD_3OD at 223K.

Figure S17. FT-IR spectrum of [Cu(L^{BSMe})$_2$] (**5**).

Figure S18. FT-IR spectrum of [Cu(HL^{BSPh})(PPh_3)$_2$]PF_6·$2CH_3CN$ (**6**).

Figure S19. ^{1}H-NMR spectrum of [Cu(L^{BSPh})(PPh)$_3$]PF_6·$2CH_3CN$ (**6**) in $CDCl_3$.

Figure S20. ^{1}H-NMR spectrum of [Cu(L^{BSPh})(PPh)$_3$]PF_6·$2CH_3CN$ (**6**) in CD_3CN.

Figure S21. ^{31}P{^{1}H}-NMR spectrum of [Cu(HL^{BSPh})(PPh_3)$_2$]PF_6·$2CH_3CN$ (**6**) in $CDCl_3$.

Figure S22. ^{31}P{^{1}H}-NMR spectrum of [Cu(HL^{BSPh})(PPh_3)$_2$]PF_6·$2CH_3CN$ (**6**) in CD_3CN.

Figure S23. FT-IR spectrum of [Cu(HL^{BSPh})(PTA)$_2$]PF_6·$2H_2O$ (**7**).

Figure S24. ^{1}H-NMR spectrum of [Cu(HL^{BSPh})(PTA)$_2$]PF_6·$2H_2O$ (**7**) in CD_3CN.

Figure S25. ^{31}P{^{1}H}-NMR spectrum of [Cu(HL^{BSPh})(PTA)$_2$]PF_6·$2H_2O$ (**7**) in CD_3CN.

Figure S26. FT-IR spectrum of [Ag(HL^{BSPh})(PPh_3)$_2$]NO_3 (**8**).

Figure S27. ^{1}H-NMR spectrum of [Ag(HL^{BSPh})(PPh_3)$_2$]NO_3 (**8**) in $CDCl_3$.

Figure S28. ^{1}H-NMR spectrum of [Ag(HL^{BSPh})(PPh_3)$_2$]NO_3 (**8**) in DMSO-d_6.

Figure S29. ^{31}P{^{1}H}-NMR spectrum of [Ag(HL^{BSPh})(PPh_3)$_2$]NO_3 (**8**) in $CDCl_3$.

Figure S30. ^{31}P{^{1}H}-NMR spectrum of [Ag(HL^{BSPh})(PPh_3)$_2$]NO_3 (**8**) in CD_3CN at 223K.

Figure S31. FT-IR spectrum of [Ag(HL^{BSPh})(PTA)]NO_3 (**9**).

Figure S32. ^{1}H-NMR spectrum of [Ag(HL^{BSPh})(PTA)]NO_3 (**9**) in DMSO-d_6.

Figure S33. ^{31}P{^{1}H}-NMR spectrum of [Ag(HL^{BSPh})(PTA)]NO_3 (**9**) in $CDCl_3$.

Figure S34. ^{31}P{^{1}H}-NMR spectrum of [Ag(HL^{BSPh})(PTA)]NO_3 (**9**) in CD_3CN at 233K.

Figure S35. FT-IR spectrum of [Cu(L^{BSPh})$_2$] (**10**).

Figure S36. Nonbonding contacts for [Cu(L^{BSPh})$_2$] (**10**); C-H····π contacts C1(C2)····H12.

Figure S37. Nonbonding contacts for [Cu(L^{BSPh})$_2$] (**10**); 2D nonbonding interaction grid formed into the *ab* plane.

Figure S38. Nonbonding contacts for [Cu(L^{BSPh})$_2$] (**10**); C-H····π interaction between H11 and the C15/C20, C15A/C20A rings; only C15····H11 approach shown.

Table S1. ^{1}H-NMR and ^{31}P{^{1}H}-NMR peaks of phenoxy-ketimine Schiff base ligands and the related Cu(I) and Ag(I) complexes recorded in CD_3CN ([a]), DMSO ([b]), CD_3OD ([c]) and $CDCl_3$ ([d]). *All spectra were recorded at 293K except for complexes **8** and **9** which were recorded at 233K.

Table S2. Bond Lengths (Å) for [Cu(L^{BSPh})$_2$] (**10**).

Table S3. Bond Angles (°) for [Cu(L^{BSPh})$_2$] (**10**).

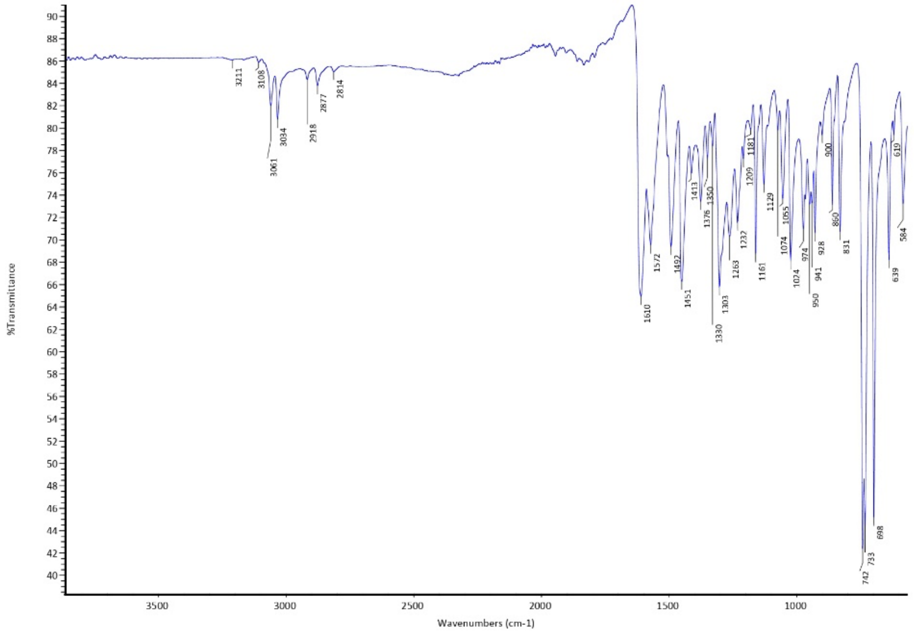

Figure S1. FT-IR spectrum of HL^{BSMe} (**1**).

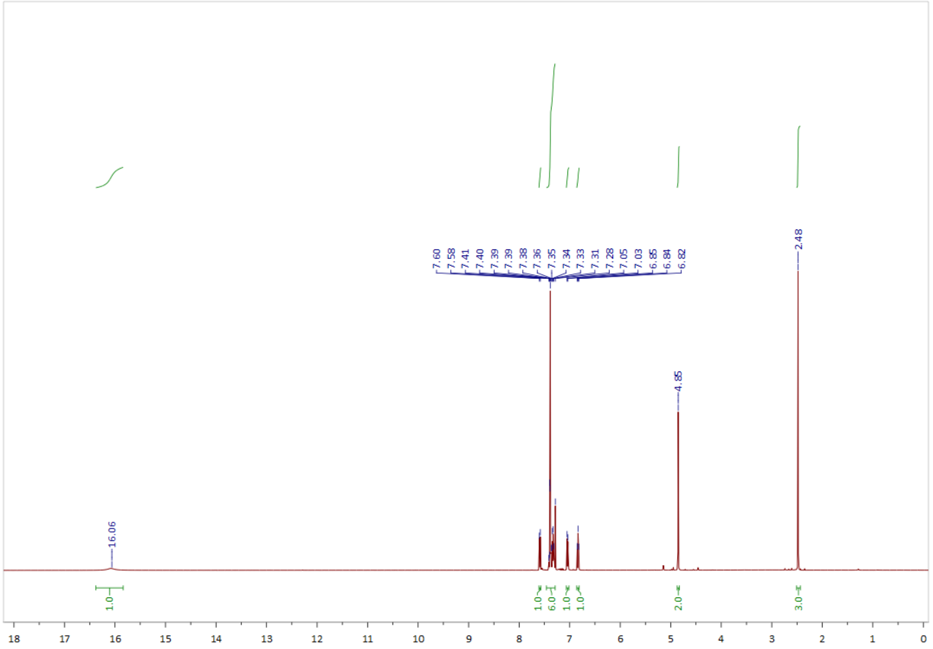

Figure S2. ^{1}H-NMR spectrum of HL^{BSMe} (**1**) in $CDCl_3$.

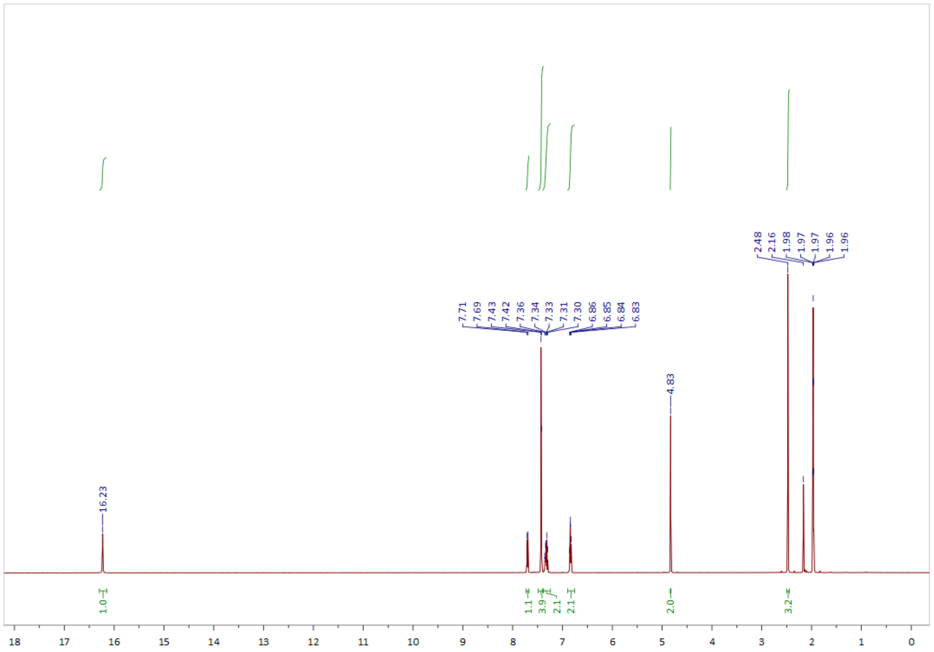

Figure S3. ^{1}H-NMR spectrum of HL^{BSMe} (**1**) in CD_3CN.

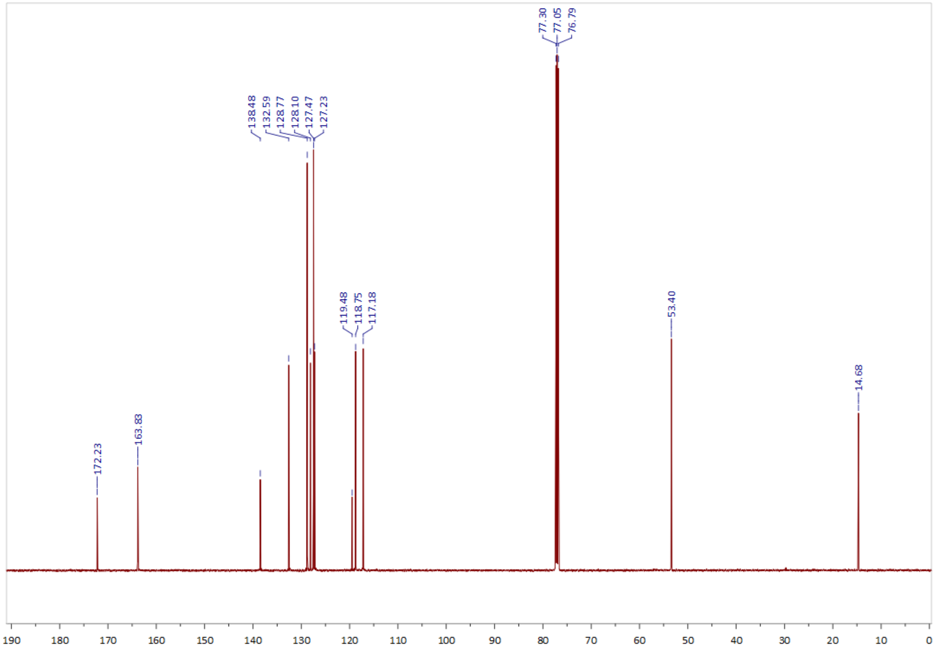

Figure S4. $^{13}C\{^{1}H\}$-NMR spectrum of HL^{BSMe} (**1**) in $CDCl_3$.

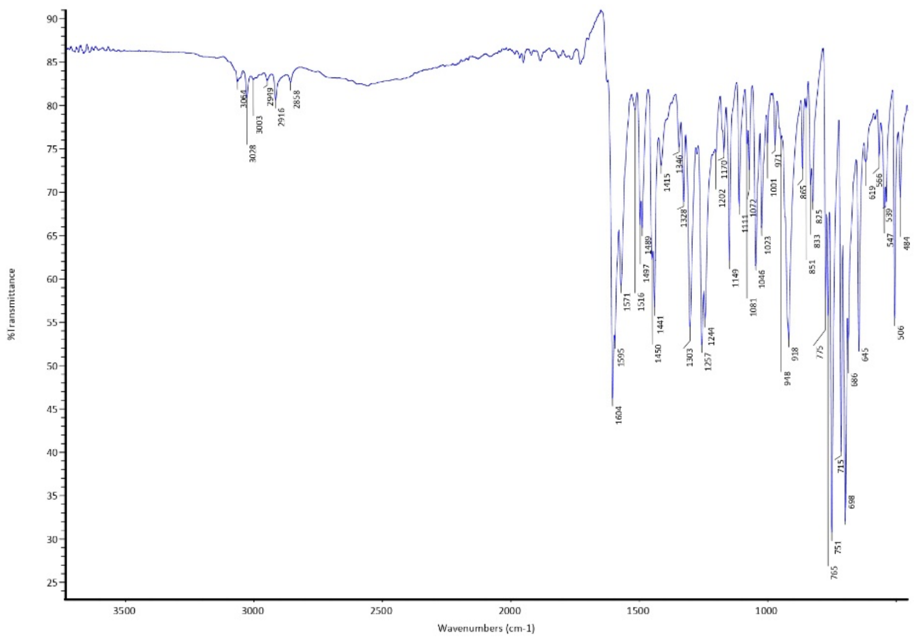

Figure S5. FT-IR spectrum of HL^{BSPh} (**2**).

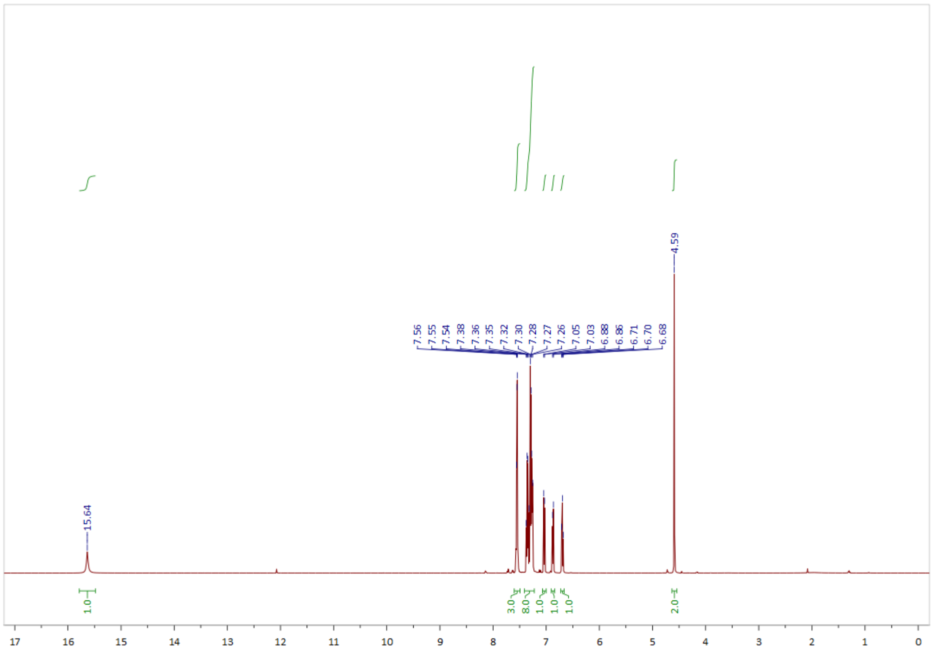

Figure S6. ^{1}H-NMR spectrum of HL^{BSPh} (**2**) in $CDCl_3$.

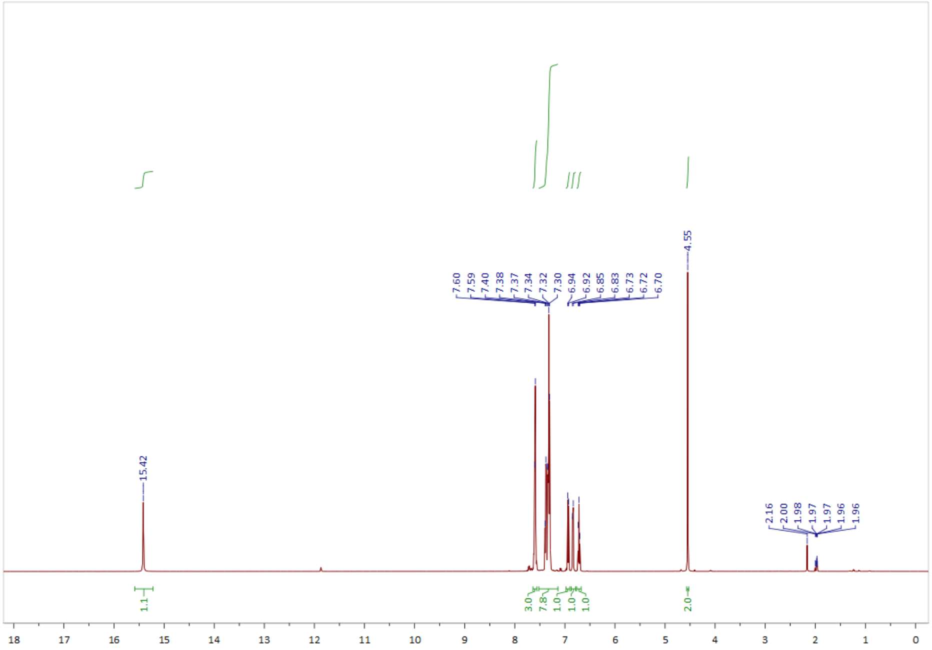

Figure S7. ^{1}H-NMR spectrum of HL^{BSPh} (**2**) in CD_3CN.

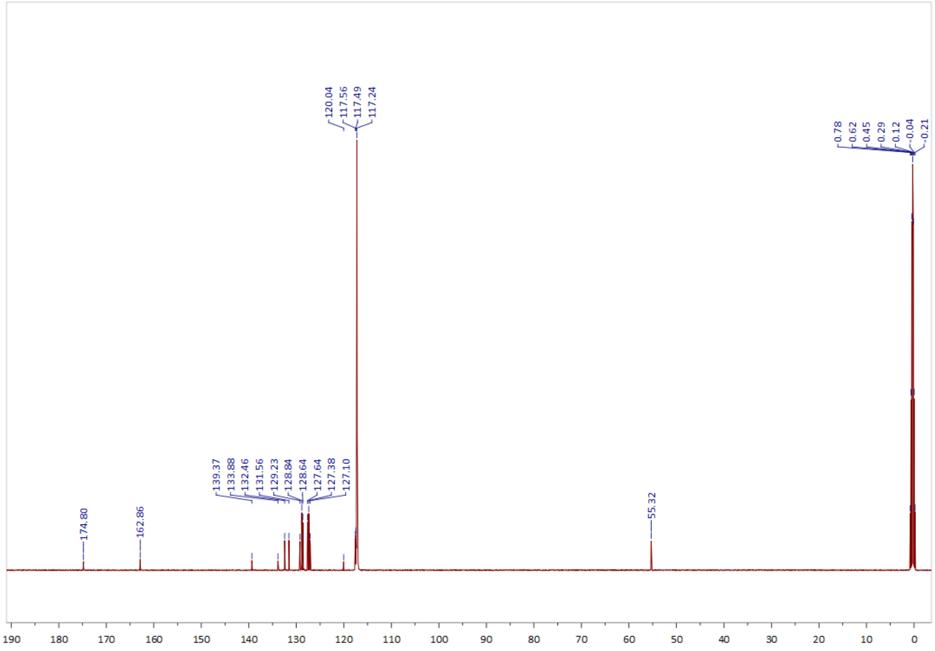

Figure S8. $^{13}C\{^{1}H\}$-NMR spectrum of HL^{BSPh} (**2**) in CD_3CN.

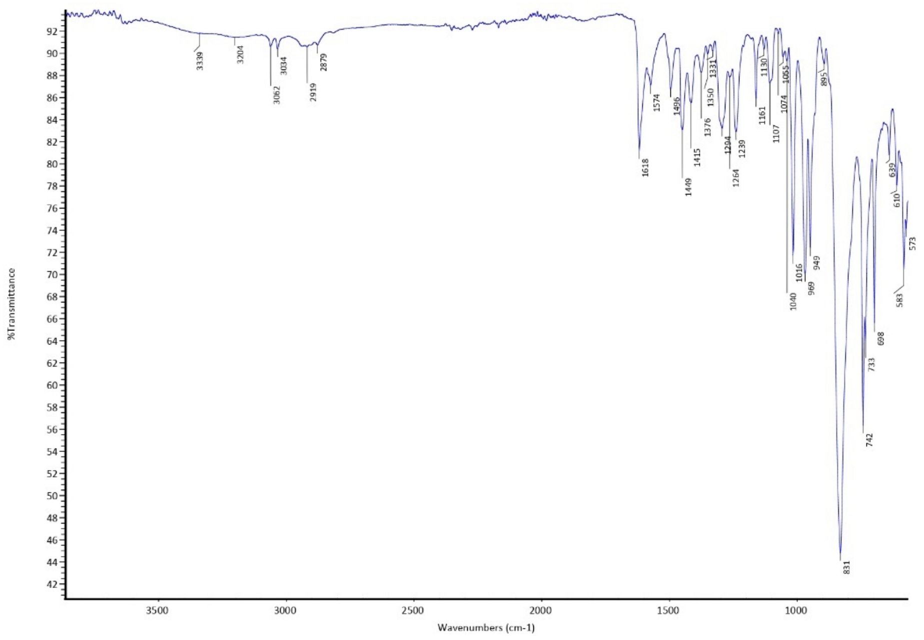

Figure S9. FT-IR spectrum of [Cu(HL^{BSMe})$(PTA)_2$]PF_6 (**3**).

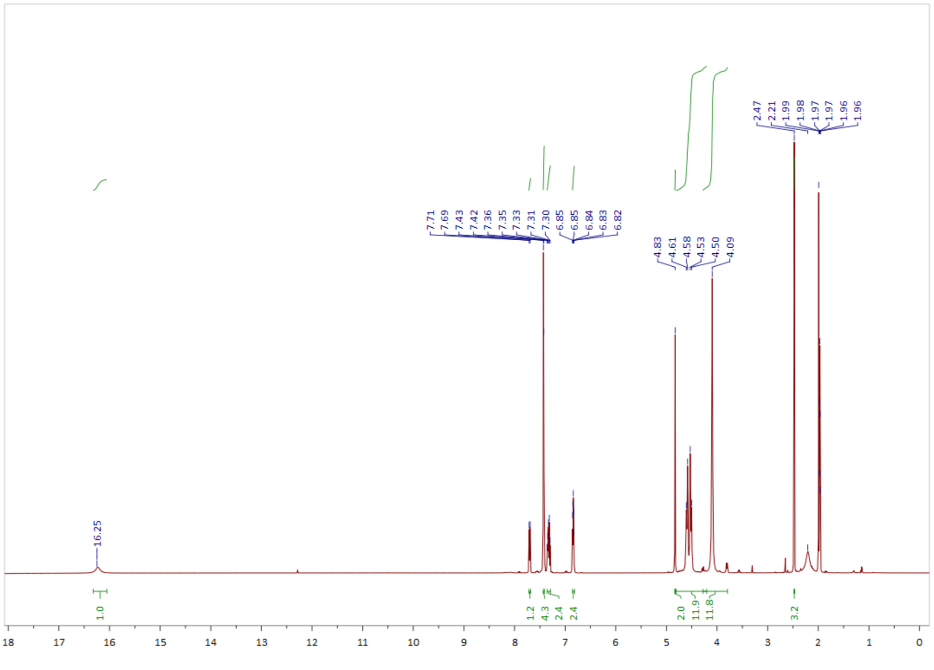

Figure S10. ^{1}H-NMR spectrum of [Cu(HL^{BSMe})$(PTA)_2$]PF_6 (**3**) in CD_3CN.

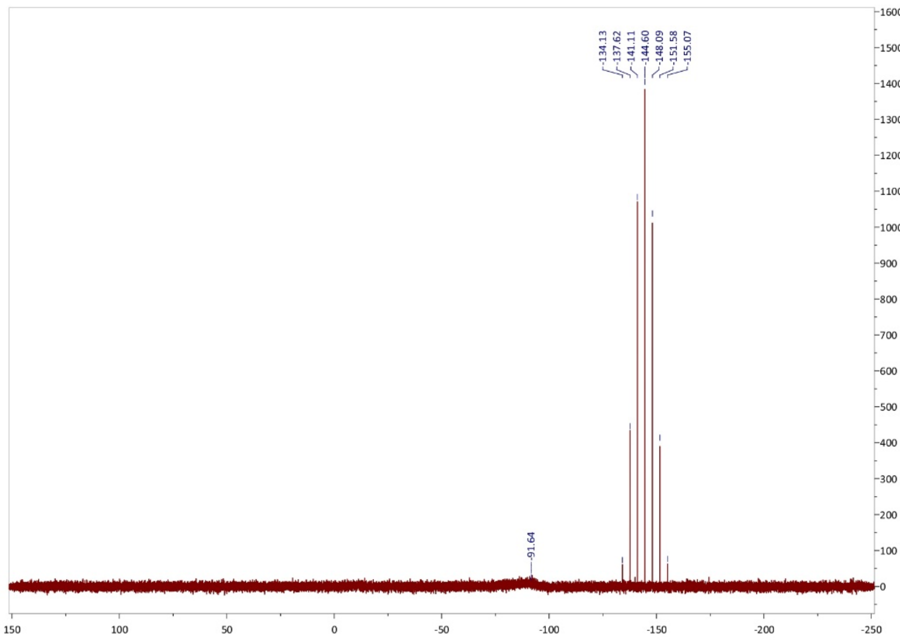

Figure S11. $^{31}P\{^{1}H\}$-NMR spectrum of $[Cu(HL^{BSMe})(PTA)_2]PF_6$ (**3**) in CD_3CN.

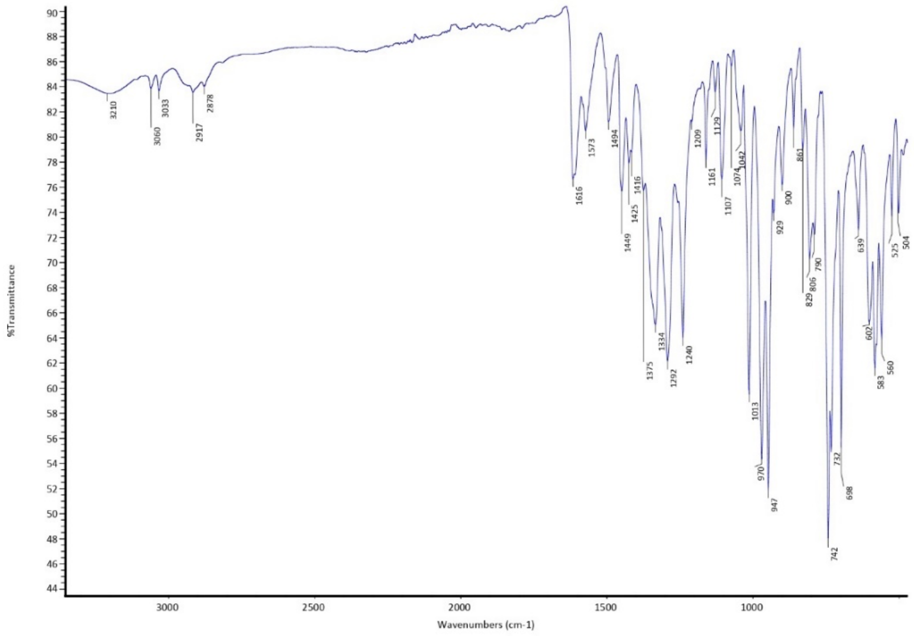

Figure S12. FT-IR spectrum of $[Ag(HL^{BSMe})(PTA)]NO_3$ (**4**).

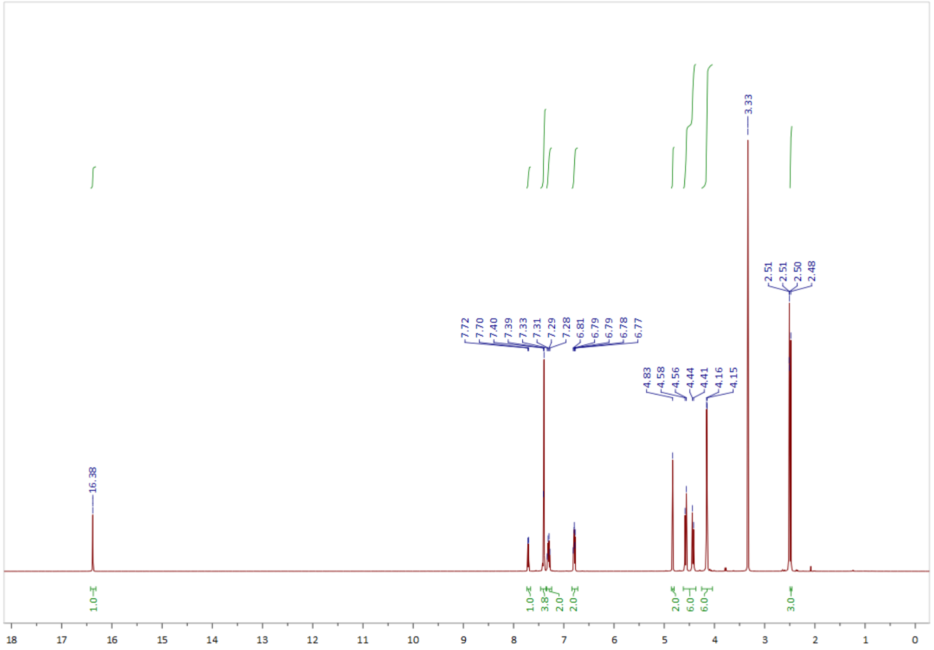

Figure S13. ^{1}H-NMR spectrum of [Ag(HL^{BSMe})(PTA)]NO_3 (**4**) in DMSO-d_6.

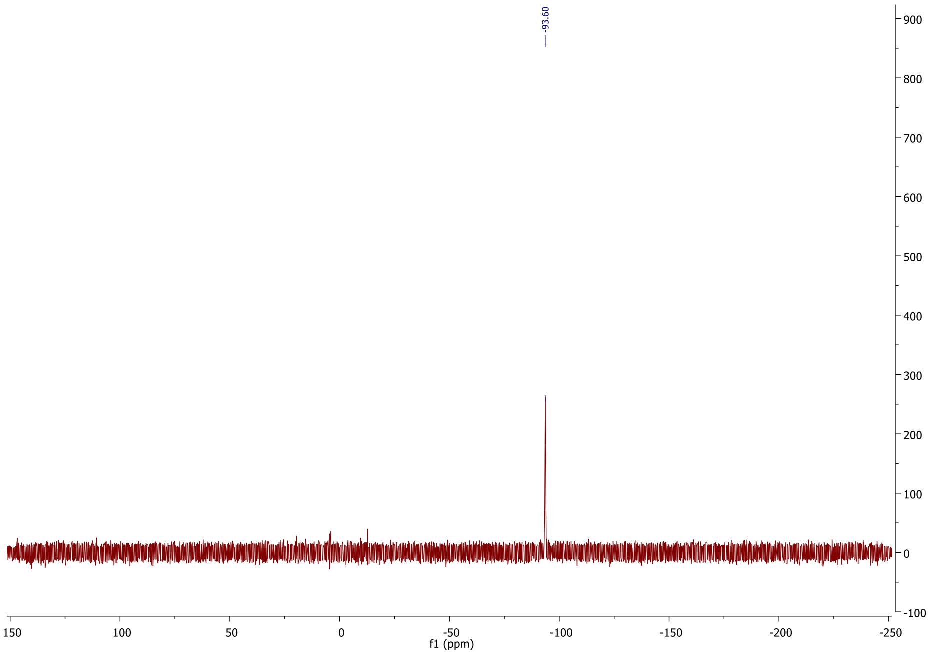

Figure S14. ^{31}P-NMR spectrum of [Ag(HL^{BSMe})(PTA)]NO_3 (**4**) in $CDCl_3$.

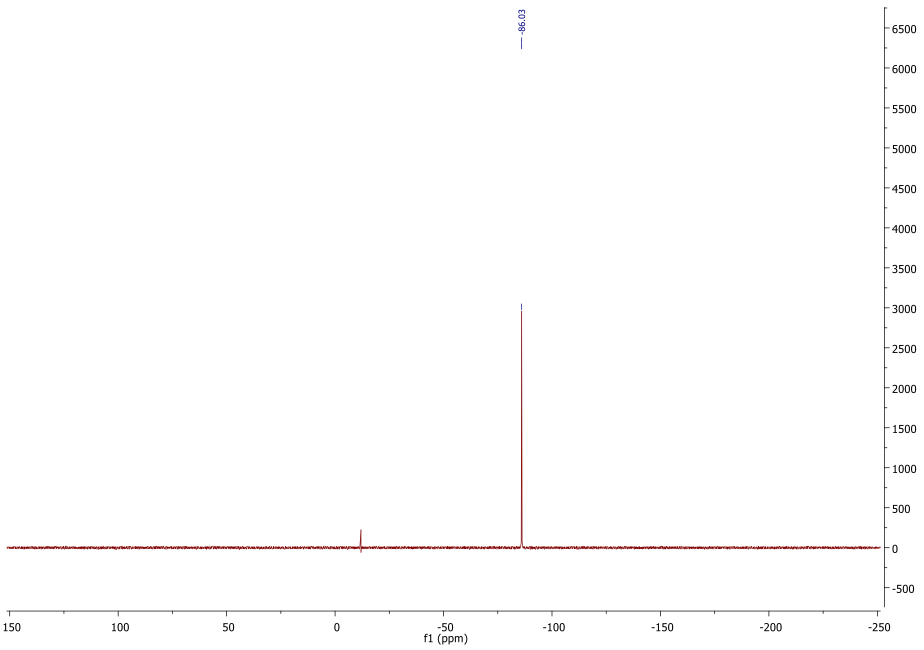

Figure S15. $^{31}P\{^{1}H\}$-NMR spectrum of $[Ag(HL^{BSMe})(PTA)]NO_3$ (**4**) in DMSO-d_6.

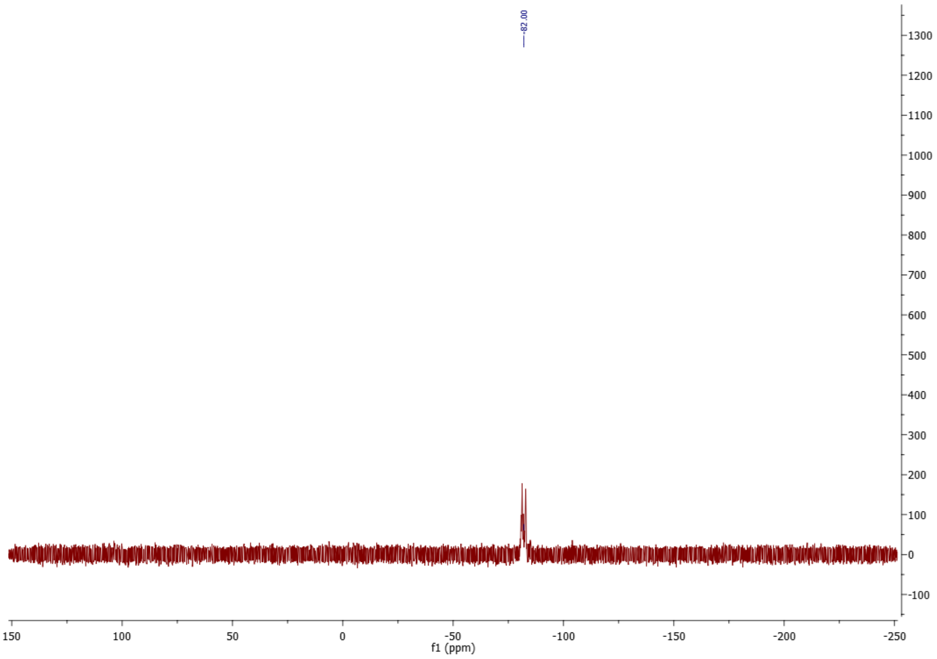

Figure S16. $^{31}P\{^{1}H\}$-NMR spectrum of $[Ag(HL^{BSMe})(PTA)]NO_3$ (**4**) in CD_3OD at 223K.

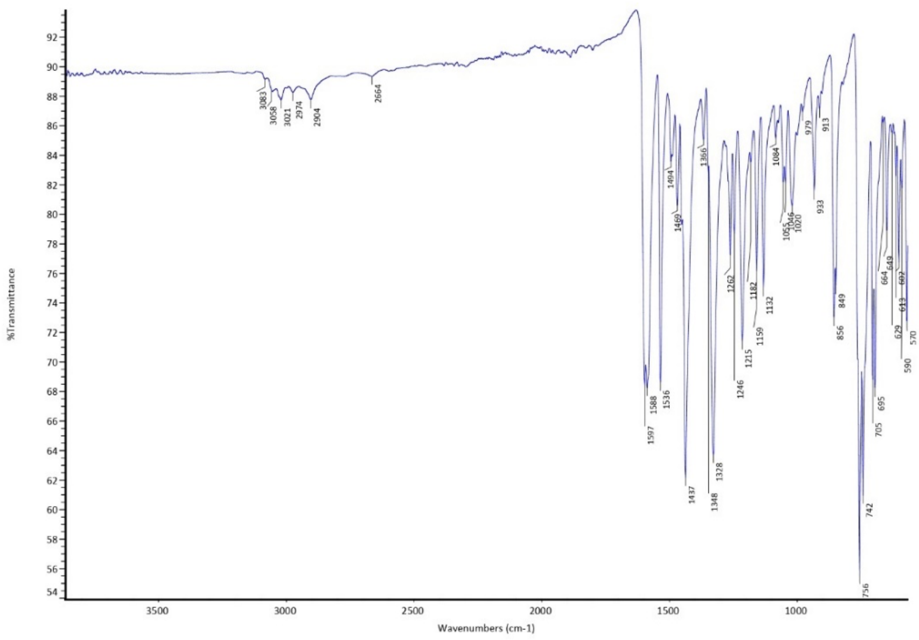

Figure S17. FT-IR spectrum of $[Cu(L^{BSMe})_2]$ (**5**).

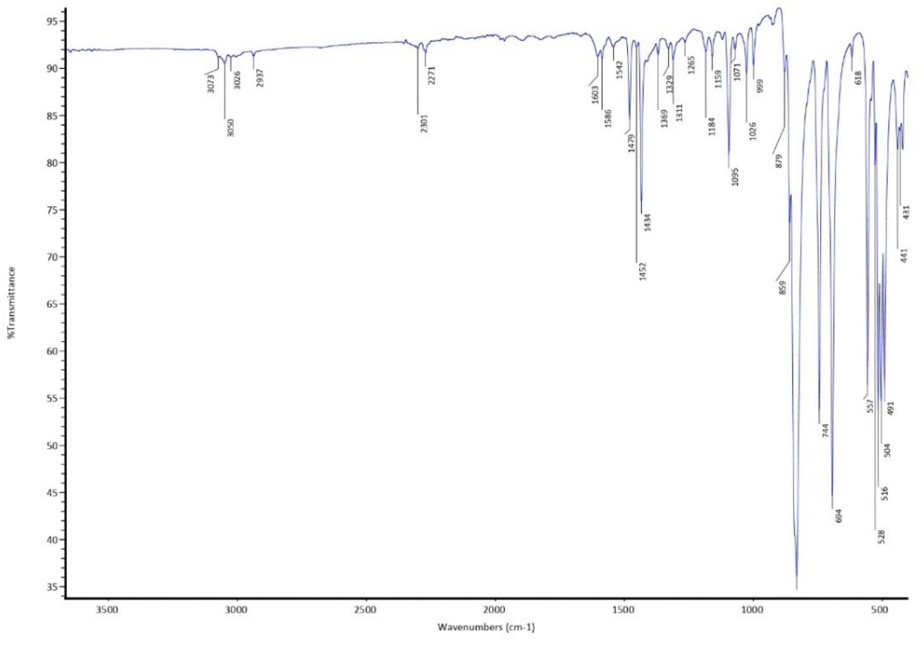

Figure S18. FT-IR spectrum of $[Cu(HL^{BSPh})(PPh_3)_2]PF_6 \cdot 2CH_3CN$ (**6**).

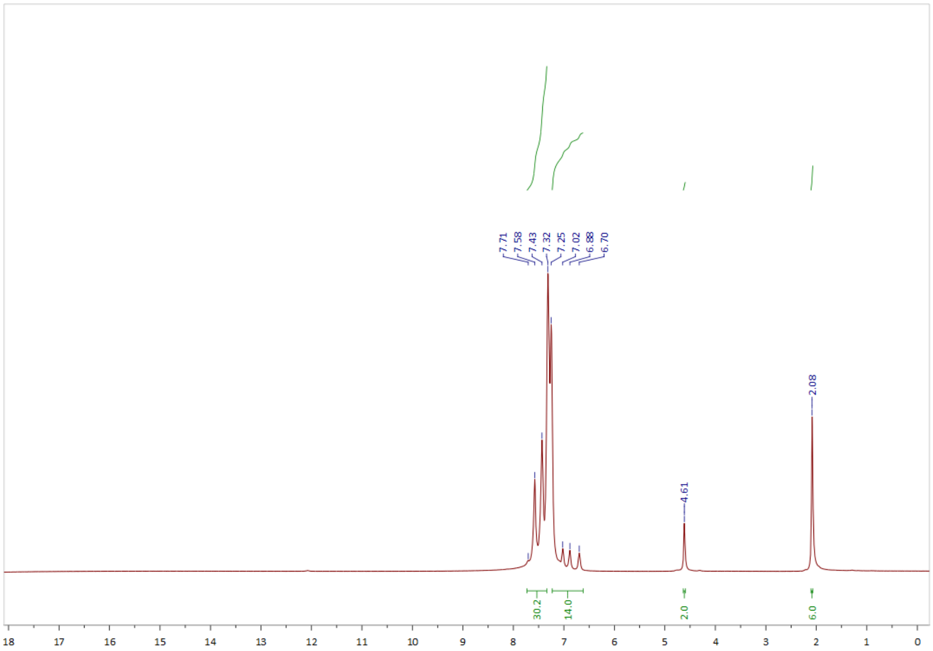

Figure S19. ^{1}H-NMR spectrum of $[Cu(L^{BSPh})(PPh)_3]PF_6{\cdot}2CH_3CN$ (**6**) in $CDCl_3$.

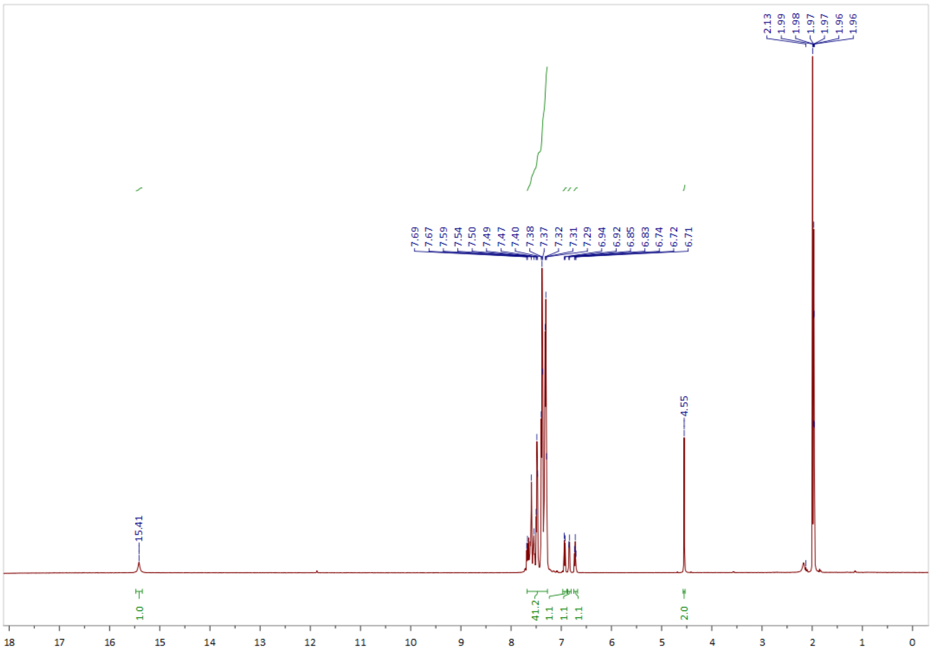

Figure S20. ^{1}H-NMR spectrum of $[Cu(HL^{BSPh})(PPh)_3]PF_6{\cdot}2CH_3CN$ (**6**) in CD_3CN.

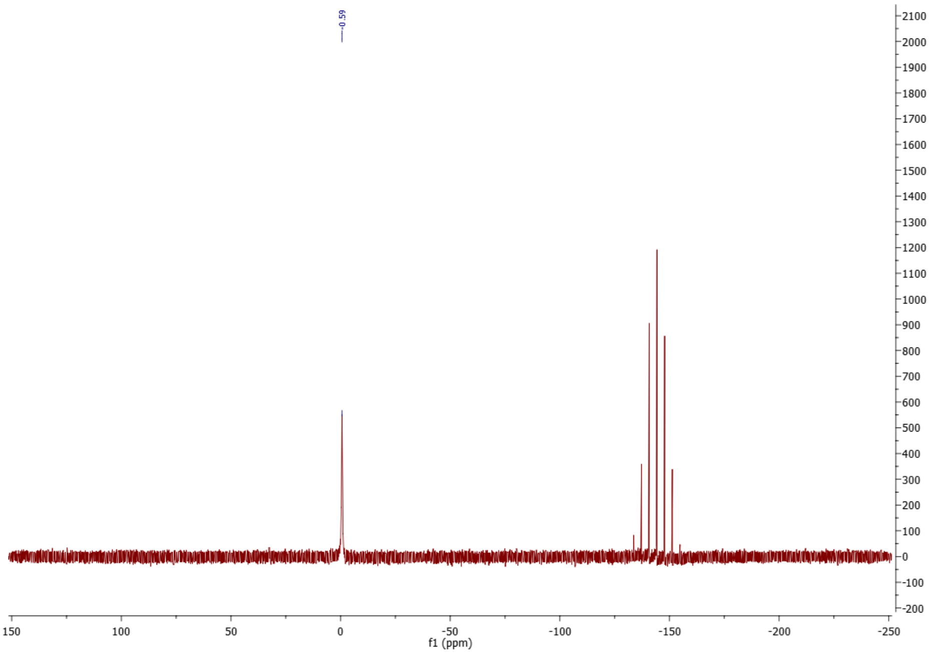

Figure S21. $^{31}P\{^{1}H\}$-NMR spectrum of $[Cu(HL^{BSPh})(PPh_3)_2]PF_6{\cdot}2CH_3CN$ (**6**) in $CDCl_3$.

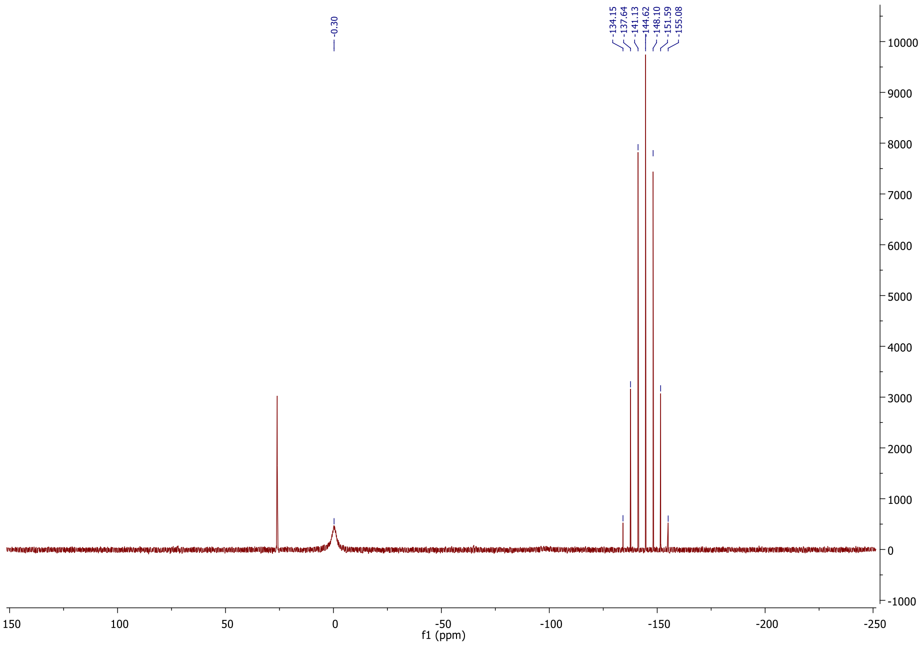

Figure S22. $^{31}P\{^{1}H\}$-NMR spectrum of $[Cu(HL^{BSPh})(PPh_3)_2]PF_6{\cdot}2CH_3CN$ (**6**) in CD_3CN.

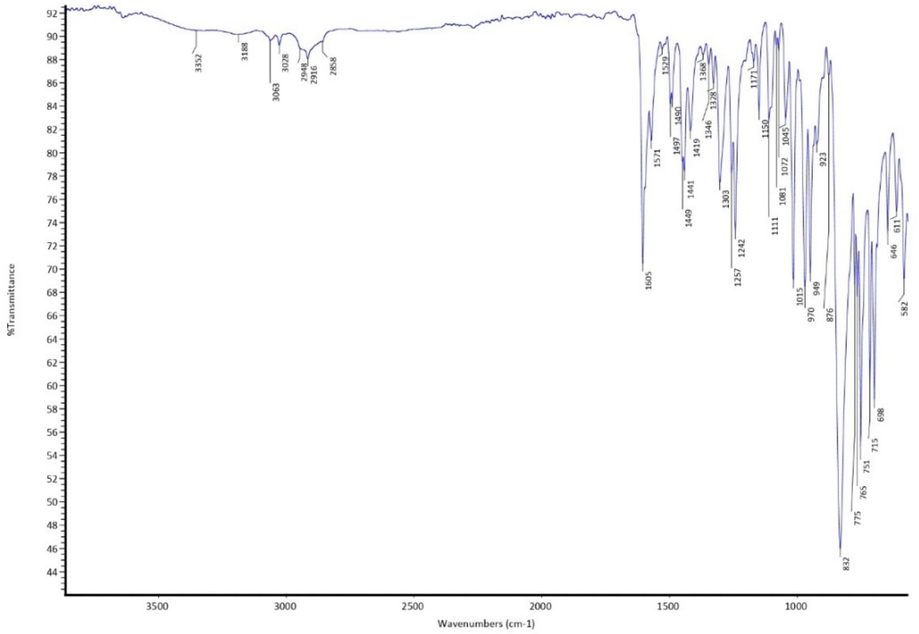

Figure S23. FT-IR spectrum of $[Cu(HL^{BSPh})(PTA)_2]PF_6{\cdot}2H_2O$ (**7**).

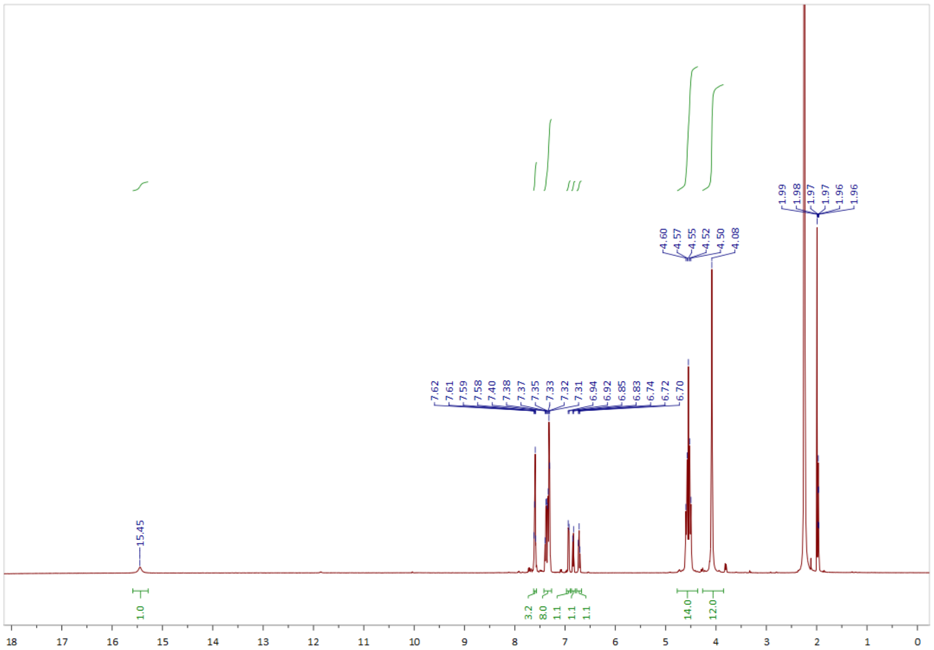

Figure S24. ^1H-NMR spectrum of $[Cu(HL^{BSPh})(PTA)_2]PF_6{\cdot}2H_2O$ in CD_3CN (**7**).

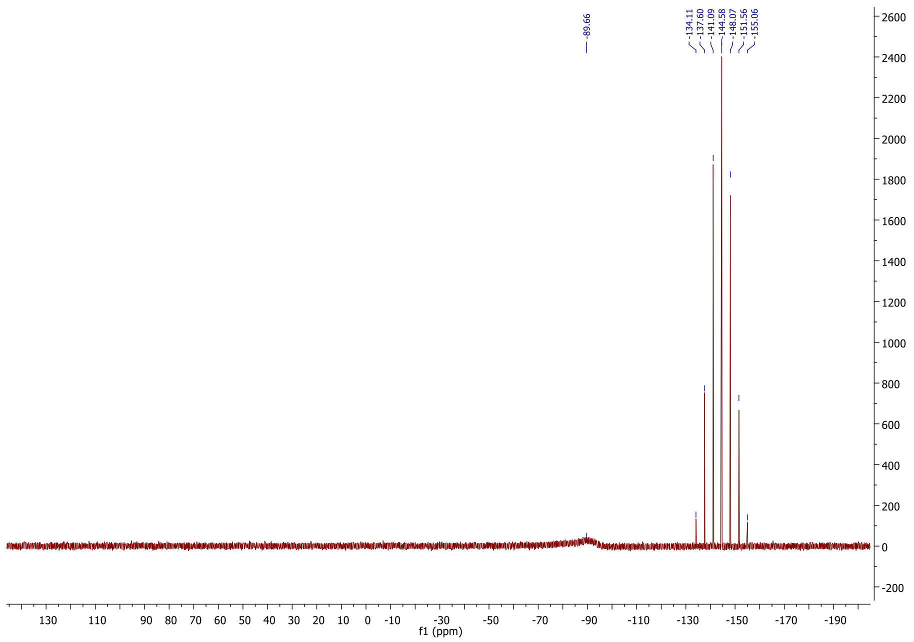

Figure S25. $^{31}P\{^1H\}$-NMR spectrum of [Cu(HL^{BSPh})(PTA)$_2$]$PF_6\cdot 2H_2O$ (**7**) in CD_3CN.

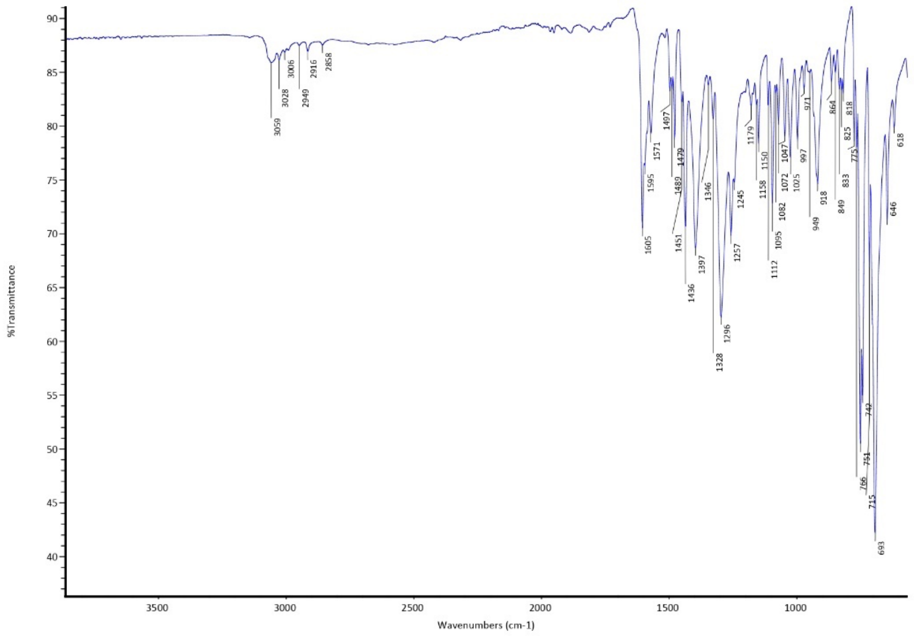

Figure S26. FT-IR spectrum of [Ag(HL^{BSPh})(PPh_3)$_2$]NO_3 (**8**).

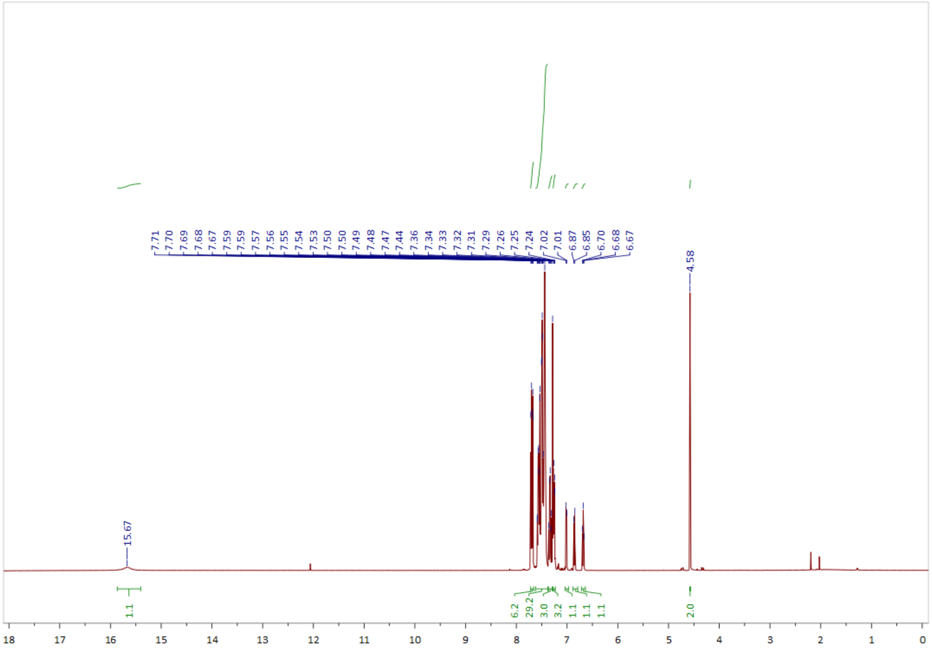

Figure S27. ^{1}H-NMR spectrum of $[Ag(HL^{BSPh})(PPh_3)_2]NO_3$ (**8**) in $CDCl_3$.

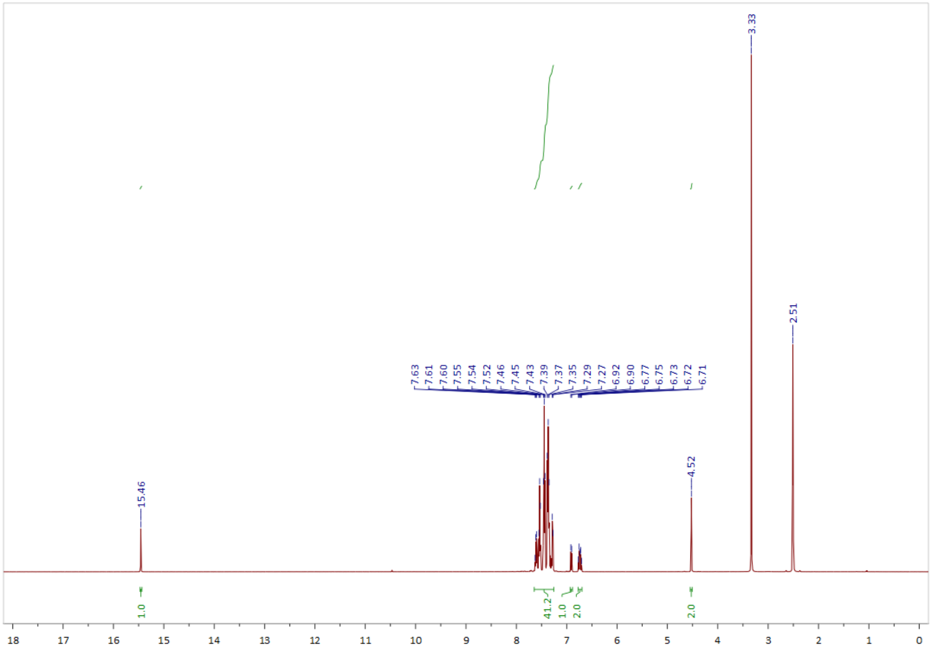

Figure S28. ^{1}H-NMR spectrum of $[Ag(HL^{BSPh})(PPh_3)_2]NO_3$ (**8**) in DMSO-d_6.

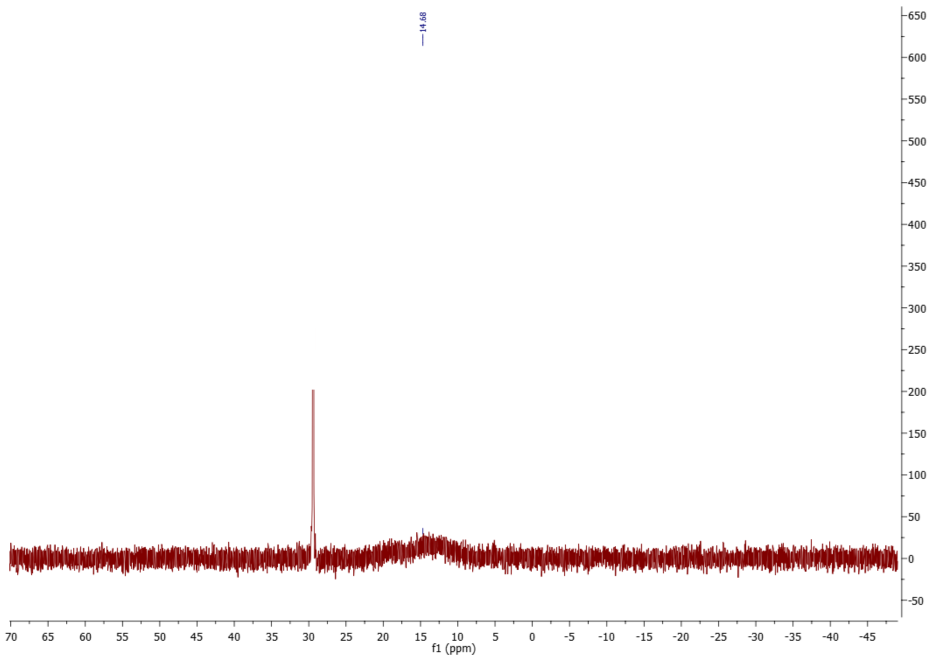

Figure S29. $^{31}P\{^{1}H\}$-NMR spectrum of $[Ag(HL^{BSPh})(PPh_3)_2]NO_3$ (**8**) in $CDCl_3$.

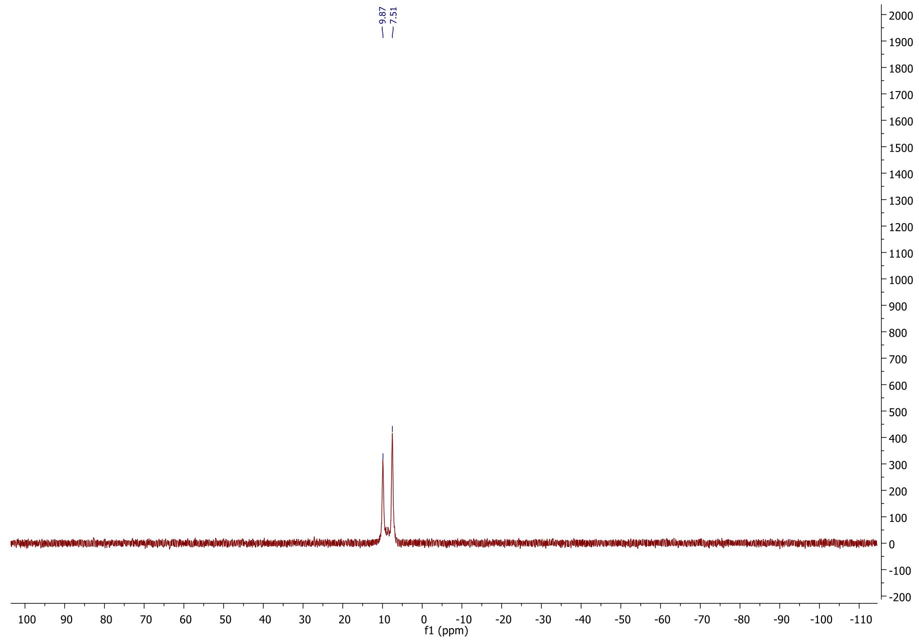

Figure S30. $^{31}P\{^{1}H\}$-NMR spectrum of $[Ag(HL^{BSPh})(PPh_3)_2]NO_3$ (**8**) in CD_3CN at 233K.

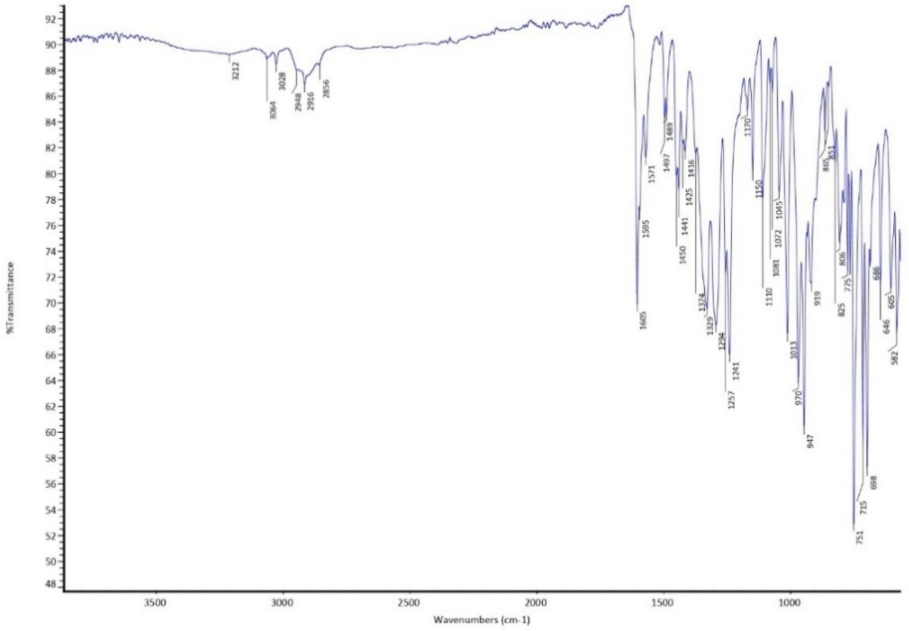

Figure S31. FT-IR spectrum of [Ag(HL^{BSPh})(PTA)]NO_3 (**9**).

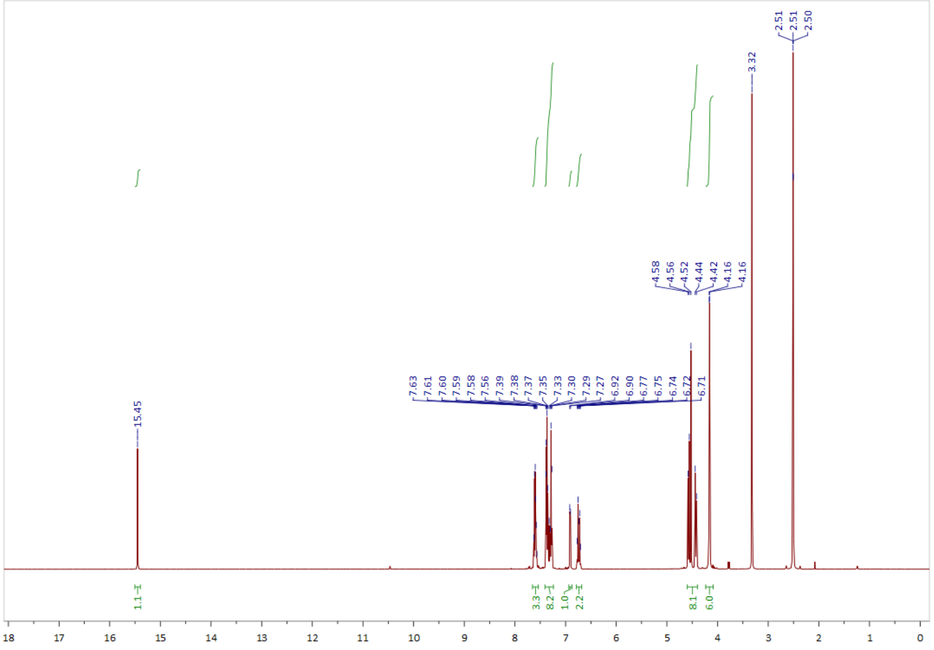

Figure S32. ^{1}H-NMR spectrum of [Ag(HL^{BSPh})(PTA)]NO_3 (**9**) in DMSO-d_6.

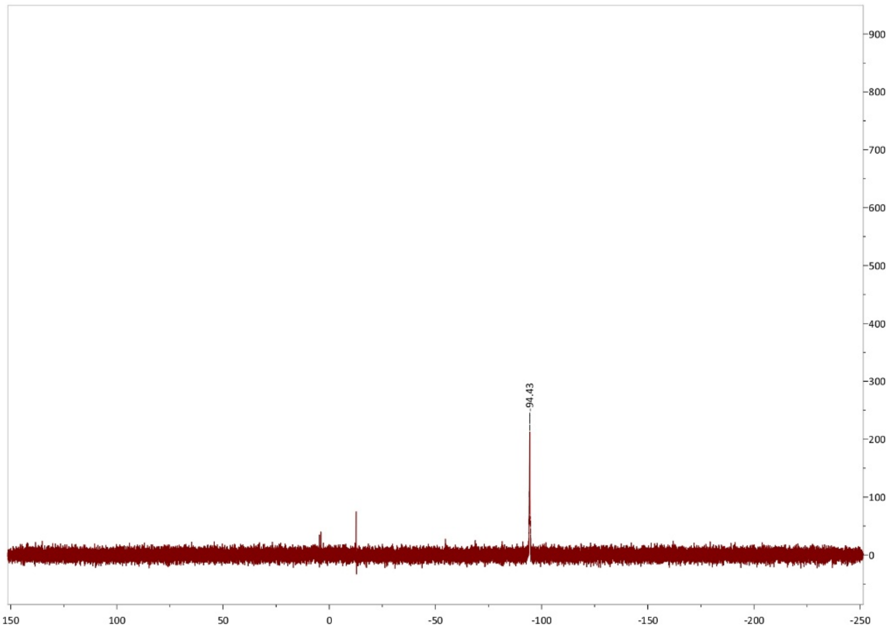

Figure S33. $^{31}P\{^{1}H\}$-NMR spectrum of $[Ag(HL^{BSPh})(PTA)]NO_3$ (**9**) in $CDCl_3$.

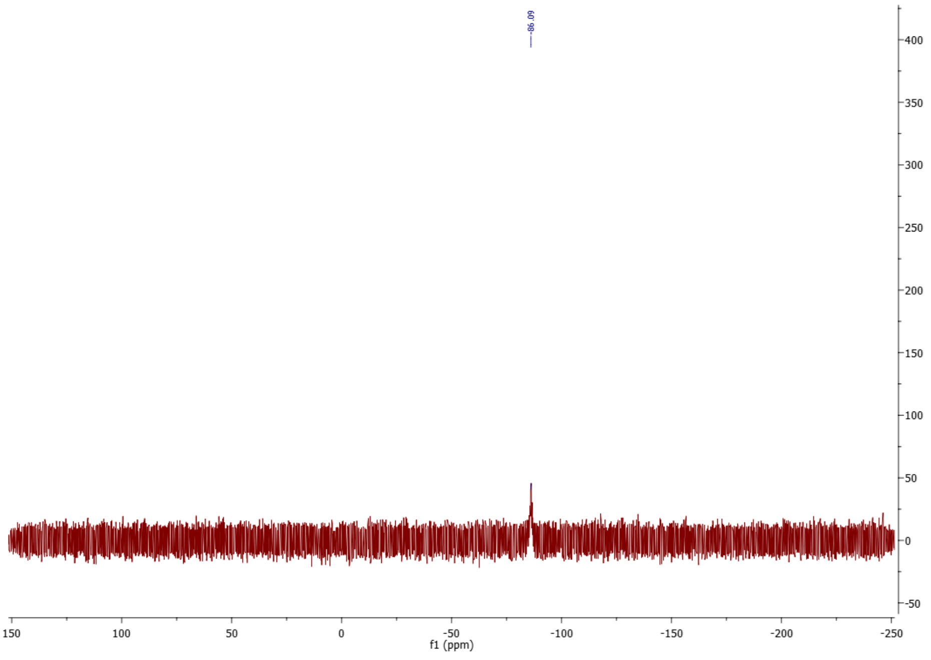

Figure S34. $^{31}P\{^{1}H\}$-NMR spectrum of $[Ag(HL^{BSPh})(PTA)]NO_3$ (**9**) in CD_3CN at 233K.

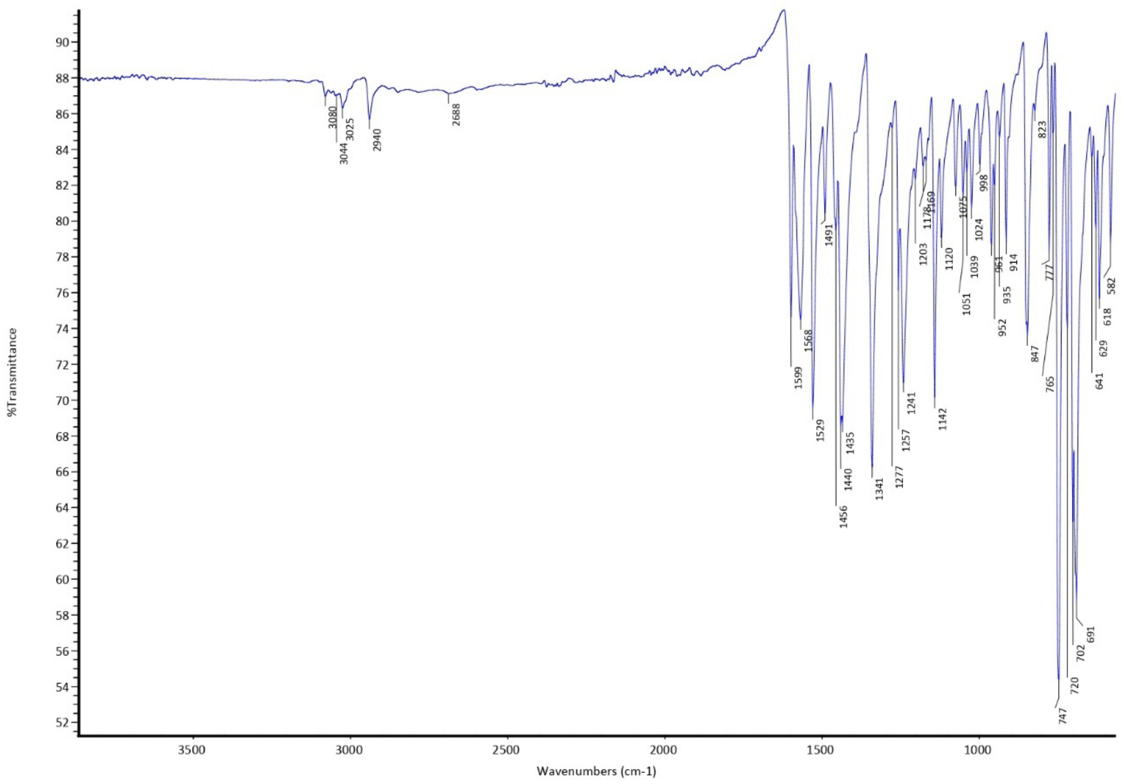

Figure S35. FT-IR spectrum of [Cu(L^{BSPh})$_2$] (**10**).

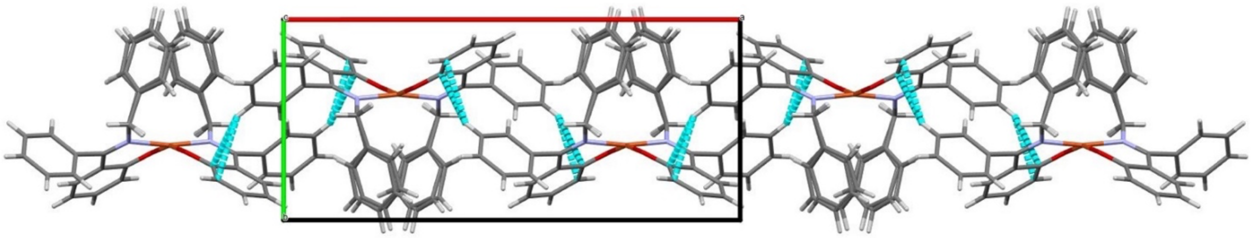

Figure S36. Nonbonding contacts for [Cu(L^{BSPh})$_2$]; projection down the crystallographic *c* axis, highlighting the head-to-tail one-dimensional chain generated by the C-H····π contacts C1(C2)····H12 (in cyan) and propagating along the crystallographic *a* axis. Other contacts omitted for clarity.

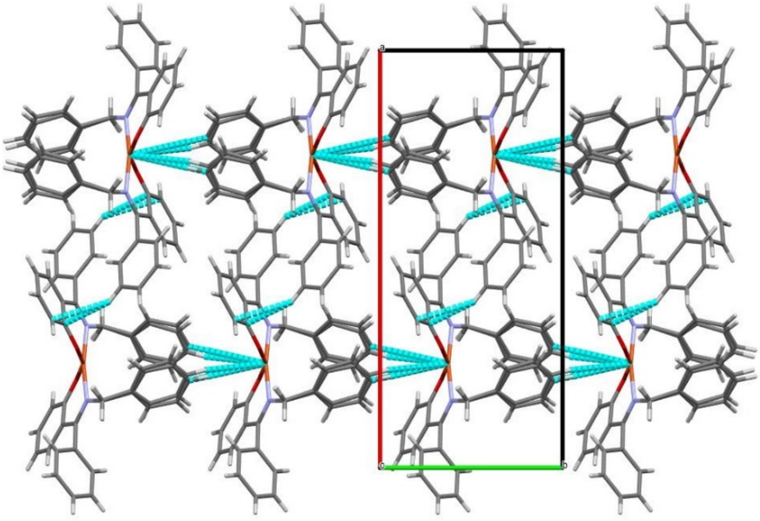

Figure S37. Nonbonding contacts for [Cu(L^{BSPh})$_2$]; projection down the crystallographic *c* axis, highlighting (in cyan) the 2D nonbonding interaction grid formed into the *ab* plane due to crosslinking between the one-dimensional motifs described in Figures 3 and S34. Other contacts omitted for clarity.

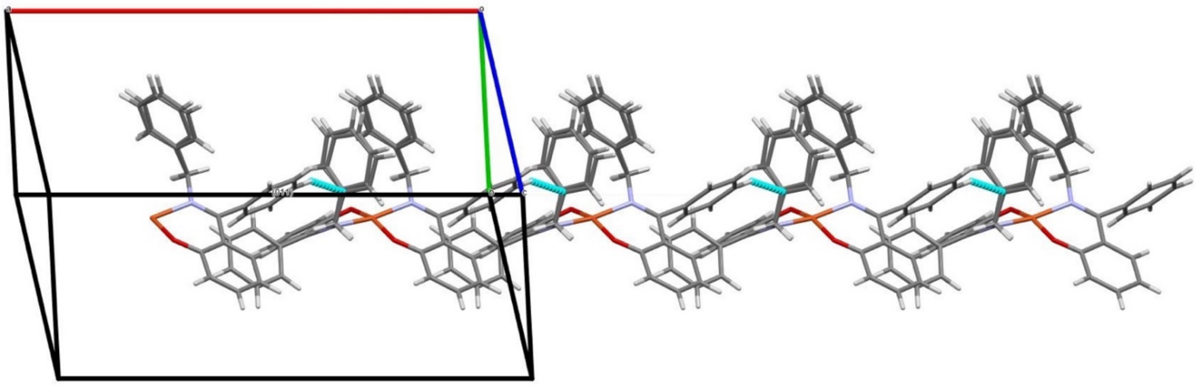

Figure S38. Nonbonding contacts for [Cu(L^{BSPh})$_2$]; projection down the [011] plane showing the one-dimensional chain generated by the (almost) orthogonal C-H····π interaction between H11 and the C15/C20, C15A/C20A rings (only the C15····H11 contact highlighted in cyan), propagating along the same plane. Other contacts omitted for clarity.

Table S1. ^{1}H-NMR and ^{31}P{^{1}H}-NMR peaks of phenoxy-ketimine Schiff base ligands and the related Cu(I) and Ag(I) complexes recorded in CD_3CN (^{a}), DMSO (^{b}), CD_3OD (^{c}) and $CDCl_3$ (^{d}). *All spectra were recorded at 293K except for complexes **8** and **9** which were recorded at 233K.

**Compounds**	δ (ppm) CH_3	δ (ppm) PTA	δ (ppm) CH_2	δ (ppm) Ar	δ (ppm) OH	δ (ppm) ^{31}P{^{1}H}
HL^{BSMe} (**1**)	2.48 s ^{a}	/	4.83 s ^{a}	6.83-7.71 m ^{a}	16.23 s ^{a}	/
HL^{BSPh} (**2**)	/	/	4.55 s ^{a}	6.70-7.60 m ^{a}	15.42 s ^{a}	/
[Cu(HL^{BSMe})$(PTA)_2$]PF_6 (**3**)	2.47 s ^{a}	4.09 s, 4.50-4.61 AB q ^{a}	4.83 s ^{a}	6.82-7.71 m ^{a}	16.25 s ^{a}	-91.64 sbr ^{a}
[Ag(HL^{BSMe})(PTA)]NO_3 (**4**)	2.48 s ^{b}	4.15 s, 4.41-4.58 AB q ^{b}	4.83 s ^{b}	6.77-7.72 m ^{b}	16.38 s ^{b}	-86.03 s ^{b} -82.00 sbr ^{c*}
[Cu(HL^{BSPh})$(PPh_3)_2$]PF_6·$2CH_3CN$ (**6**)	/	/	4.55 s ^{a}	6.71-7.69 m ^{a}	15.41 s ^{a}	-0.30 sbr ^{a}
[Cu(HL^{BSPh})$(PTA)_2$]PF_6·$2H_2O$ (**7**)	/	4.08 s, 4.50-4.60 AB q ^{a}	4.55 s ^{a}	6.70-7.62 m ^{a}	15.45 s ^{a}	-89.66 sbr ^{a}
[Ag(HL^{BSPh})$(PPh_3)_2$]NO_3 (**8**)	/	/	4.52 s ^{b}	6.71-7.63 m ^{b}	15.46 s ^{b}	8.69 d (J(Ag-^{31}P) = 478 Hz) ^{a*}
[Ag(HL^{BSPh})(PTA)]NO_3 (**9**)	/	4.16 s, 4.42-4.58 AB q ^{b}	4.52 s ^{b}	6.71-7.63 m ^{b}	15.45 s ^{b}	-94.43 s ^{d} -86.09 sbr ^{a*}

Table S2. Bond Lengths (Å) for [Cu(L^{BSPh})$_2$] (**10**).

Atom	Atom		Atom	Atom	
Cu1	O1	1.8839(11)	C10	C11	1.376(3)
Cu1	O1[1]	1.8839(11)	C11	C12	1.370(3)
Cu1	N1[1]	1.9773(12)	C12	C13	1.385(3)
Cu1	N1	1.9773(12)	C14	C15	1.483(10)
O1	C1	1.303(2)	C14	C15A	1.536(11)
N1	C7	1.299(2)	C15	C16	1.360(8)
N1	C14	1.4787(17)	C15	C20	1.369(8)
C1	C2	1.420(2)	C16	C17	1.382(9)
C1	C6	1.417(2)	C17	C18	1.347(12)
C2	C3	1.363(3)	C18	C19	1.322(13)
C3	C4	1.384(3)	C19	C20	1.386(8)
C4	C5	1.373(2)	C20A	C19A	1.3900
C5	C6	1.412(2)	C20A	C15A	1.3900
C6	C7	1.4654(19)	C19A	C18A	1.3900
C7	C8	1.5009(19)	C18A	C17A	1.3900
C8	C9	1.390(2)	C17A	C16A	1.3900
C8	C13	1.390(2)	C16A	C15A	1.3900
C9	C10	1.380(2)			

[1]symmetry operation: 3/2 – *x*, *y*, 1 – *z*

Table S3. Bond Angles (°) for $[Cu(L^{BSPh})_2]$ (**10**).

**Atom**	**Atom**	**Atom**		**Atom**	**Atom**	**Atom**	
O1	Cu1	O1[1]	160.77(9)	C13	C8	C9	118.83(14)
O1[1]	Cu1	N1	90.20(5)	C10	C9	C8	120.10(16)
O1	Cu1	N1[1]	90.20(5)	C11	C10	C9	120.77(16)
O1[1]	Cu1	N1[1]	91.46(5)	C12	C11	C10	119.51(17)
O1	Cu1	N1	91.46(5)	C11	C12	C13	120.53(19)
N1[1]	Cu1	N1	170.07(7)	C12	C13	C8	120.25(17)
C1	O1	Cu1	124.80(10)	N1	C14	C15	113.9(7)
C7	N1	Cu1	125.32(10)	N1	C14	C15A	108.1(7)
C7	N1	C14	120.91(12)	C16	C15	C14	122.7(9)
C14	N1	Cu1	113.63(9)	C16	C15	C20	116.7(7)
O1	C1	C2	117.14(14)	C20	C15	C14	120.6(9)
O1	C1	C6	124.98(13)	C15	C16	C17	121.4(8)
C6	C1	C2	117.88(14)	C18	C17	C16	120.1(9)
C3	C2	C1	121.81(16)	C19	C18	C17	119.9(6)
C2	C3	C4	120.51(15)	C18	C19	C20	120.5(7)
C5	C4	C3	119.43(17)	C15	C20	C19	121.3(7)
C4	C5	C6	122.06(16)	C19A	C20A	C15A	120.0
C1	C6	C7	122.71(13)	C18A	C19A	C20A	120.0
C5	C6	C1	118.30(13)	C19A	C18A	C17A	120.0
C5	C6	C7	118.95(13)	C16A	C17A	C18A	120.0
N1	C7	C6	122.33(13)	C17A	C16A	C15A	120.0
N1	C7	C8	120.34(12)	C20A	C15A	C14	120.9(8)
C6	C7	C8	117.32(12)	C16A	C15A	C14	118.6(8)
C9	C8	C7	119.69(14)	C16A	C15A	C20A	120.0
C13	C8	C7	121.47(13)				

[1]symmetry operation: 3/2 – *x*, *y*, 1 – *z*
